# A TROP2-targeting ADC synergizes with oxidative phosphorylation inhibitor to enhance apoptosis in ESCC by suppressing the PI3K-AKT-mTOR signaling pathway

**DOI:** 10.1038/s41419-025-08278-5

**Published:** 2025-12-01

**Authors:** Xinxin Liu, Jie Liu, Yi’an Zhou, Mei Li, Feiqi Wang, Guanfeng Jiang, Youban Xiao, Wenming Cui, Mingfeng Jiang, Liang Gu, Zuxiong Zhang, Yong Zheng, Shuyong Zhang

**Affiliations:** 1 Department of Respiratory and Critical Care Medicine, The First Affiliated Hospital, Gannan Medical University, Ganzhou, 341000 Jiangxi China; 2 Key Laboratory of Prevention and Treatment of Cardiovascular and Cerebrovascular Diseases (Ministry of Education), Gannan Medical University, Ganzhou, 341000 Jiangxi China; 3 Gannan Branch of National Clinical Research Center for Geriatrics, Ganzhou, 341000 Jiangxi China; 4 School of Rehabilitation Medicine, Gannan Medical University, Ganzhou, 341000 Jiangxi China; 5 School of Medical Information Engineering, Gannan Medical University, Ganzhou, 341000 Jiangxi China; 6 School of Basic Medicine, Gannan Medical University, Ganzhou, 341000 Jiangxi China; 7 Department of Thoracic Surgery, The First Affiliated Hospital, Gannan Medical University, Ganzhou, 341000 Jiangxi China

**Keywords:** Pharmacology, Cancer therapy

## Abstract

Currently, there are no effective targeted therapies for advanced esophageal squamous cell carcinoma (ESCC). Trophoblast cell surface antigen-2 (TROP2) are considered robust therapeutic targets which leverages antibody-drug conjugates (ADCs) to control solid tumors. Mitochondrial oxidative phosphorylation (OXPHOS) influences the growth of cancer cells, metastasis, and drug resistance. However, whether inhibiting OXPHOS can potentiate the efficacy of TROP2-targeting ADCs is not well understood. Here, we investigated the therapeutic efficacy of IMMU132 (IMMU), an ADC targeting TROP2, either alone or in combination with IACS010759 (IACS), a selective OXPHOS inhibitor, through clinically ESCC models. Immunohistochemical analysis was performed on a cohort of 222 patients, which revealed that 94.6% of all specimens tested positive for TROP2. Among them, moderate and strong staining were observed in 29.3% and 27.0% of cases, respectively. Co-administration of IMMU and IACS synergistically inhibited the growth of tumors in human ESCC cell lines, PDXO, and PDX models. Mechanistically, we found that the combination treatment achieved tumor suppression in ESCC cells via inducing apoptosis and oxidative stress, as well as preventing cell motility. Results of the RNA-seq analysis demonstrated that the combined treatment of IMMU and IACS downregulated the expression level of several cancer-related pathways, such as the PI3K-AKT-mTOR pathway, OXPHOS, and apoptosis. Moreover, the data confirmed that inhibition of the PI3K-AKT-mTOR pathway significantly suppressed ESCC tumor growth following administration of the combination therapy. Based on these findings, we present a novel therapeutic strategy that enhances the efficacy of TROP2-targeting ADCs via concurrent inhibition of OXPHOS, which is likely to improve clinical outcomes of patients with TROP2-positive ESCC.

## Introduction

Esophageal cancer is an aggressive gastrointestinal malignancy, that is prevalent worldwide. The cancer is classified into esophageal squamous cell carcinoma (ESCC) and esophageal adenocarcinoma (EAC) [1, 2]. ESCC is the predominant subtype in China [3], which is primarily treated with surgical resection, combined with chemotherapy or radiation therapy as adjunctive measures. Although several therapies have been developed, the overall 5-year survival rate of patients with ESCC is less than 20% [4]. Moreover, severe side effects associated with radiation therapy and chemotherapy may aggravate the patient’s already poor prognosis [5]. This calls for the design of effective strategies to improve the prognosis of ESCC patients.

Antibody-drug conjugates (ADCs) are considered effective therapeutics for the management of solid and hematological malignancies owing to their capacity to synergize with cytotoxic payloads for targeted tumoricidal delivery [6–8]. ADC therapies targeted EGFR, glypican-1, PIEZO1 for ESCC have been reported in various preclinical investigations [9–11]. However, the demand for novel and effective therapeutic strategies for ESCC treatment is huge.

Trophoblast cell surface antigen-2 (TROP2), a type I cell surface glycoprotein, has been recognized as a pan-cancer target which is overexpressed in most solid tumors, and its overexpression has been linked to poor prognosis and increased risk of metastasis [12–14]. Therefore, TROP2 is postulated to be a potential and promising target in several human solid tumor. Currently, several TROP2-targeting ADCs are used to treat various tumors. Sacituzumab govitecan (IMMU132, IMMU), a first-in-class ADC comprising a humanized IgG1κ anti-TROP2 antibody attached to the active metabolite of irinotecan (SN-38) via a hydrolyzable CL2A linker, was approved for the clinical management of TROP2-positive, unresectable, locally advanced or metastatic triple negative breast cancer (TNBC) and urothelial carcinoma (UC) [14, 15]. Other researchers have tested the use of IMMU132-01 in a phase I/II basket trial in terms of its safety and efficacy in patients with advanced solid tumors, including UC, breast cancer (BC), TNBC, colorectal cancer (CRC), non-small cell lung cancer (NSCLC), small cell lung cancer (SCLC), castration-resistant prostate cancer, esophageal carcinoma and endometrial carcinoma [16–22]. Nevertheless, there is limited data on the effectiveness of IMMU in advanced and metastatic esophageal cancer [23]. Elsewhere, the preclinical studies by Hoppe et al. demonstrated that IMMU can potentially treat EAC [24]. This implies that IMMU, either alone or in combination with other therapies, may have beneficial effects on TROP2-positive ESCC.

Notably, the metabolic characteristics of tumor cells undergo numerous changes during progression as they adapt to the microenvironment and rapid growth patterns. Several investigations have shown that the mitochondrial aerobic respiration and oxidative phosphorylation (OXPHOS) are major processes that serve to maintain the sustained proliferation of cancer cells, which are mostly likely to influence the occurrence of drug resistance in malignant tumors. Evidence from recent studies shows that specific tumors, such as BC [25], recurrent acute myeloid leukemia (AML) [26] and pancreatic cancer [27], mainly rely on OXPHOS to sustain their proliferation and survival. Notably, chemotherapy-resistant tumor stem cells and tumor cells which are resistant to targeted therapies, whether primary or acquired, largely rely on OXPHOS [28, 29]. Indeed, inhibition of the OXPHOS complexes can interfere with the respiratory pathway of tumor cells, thereby suppressing the biomass generation and ultimately inducing cell death. Currently, researchers utilize three strategies to develop OXPHOS inhibitors, i.e., inhibition of mitochondrial function, suppression of the respiratory chain complexes, and inhibition of several key targets associated with the OXPHOS. Metformin, a complex I inhibitor, was approved for the treatment of type-2 diabetes and is the most prescribed drug worldwide. It is undergoing clinical trials for potential application in the clinical control of CRC, and BC [30, 31]. Previously, Stephenson et al. found that carboxymethyl triazole could inhibit the activity of mitochondrial complex I and decrease the level of OXPHOS in tumor cells, thereby suppressing the proliferation of tumor cells [32]. Additionally, IACS010759 (IACS), a selective and less toxic inhibitor of complex I, exhibited good tolerability in diverse clinical trials for AML and specific solid tumors [33]. Based on this background, targeting OXPHOS and its combination therapy is postulated to be a robust strategy for cancer treatment.

In this study, we aimed to systematically explore the feasibility of combining TROP2-targeting ADC (IMMU) and OXPHOS inhibitor (IACS) to treat patients with advanced ESCC. The data uncovered that the combination of IMMU and IACS synergistically induced apoptosis in vitro in TROP2-positive ESCC cells. It also inhibited the growth of ESCC organoids ex vivo and PDX tumors in vivo. Further analysis confirmed that the combination suppressed the ESCC growth by targeting the PI3K-AKT-mTOR signaling pathway. In summary, these findings provide robust preclinical evidence to support the therapeutic benefits of OXPHOS inhibitors in potentiating the efficacy of TROP2-targeting ADCs in patients with ESCC.

## Methods and materials

### Reagents and antibodies

IMMU (S22F026D) was purchased from Gilead Sciences, Inc (California, USA). IACS (S8731), Rapamycin (S1039), and MHY1485 (S7811) were purchased from Selleck (Houston, USA). Antibody tested in this study was listed in Supplementary Table 1.

### Cell lines and cell culture

Several ESCC cell lines, including KYSE30 (#20210720), KYSE150 (#20210815), KYSE410 (#20210820), and TE-12(#20210901), were bought from the cell iCell Bioscience, Inc (Shanghai, China). They were identified through short tandem repeats (STR) and mycoplasma contamination was ruled out. KYSE30 and KYSE150 cells were cultured in a medium containing RPMI-1640 and F12 medium (Gibco) in a 1:1 ratio with 1% glutamine (Gibco). KYSE410 cells were cultured in RPMI-1640 medium (Gibco). TE-12 cells were cultured in DMEM medium (Sigma). The culture medium of all cells contained 10% fetal bovine serum (FBS, Gibco) and 1% penicillin/streptomycin (Biochem, Shenzhen, China). All cell lines were cultured in a humidified incubator (Thermo Fisher Scientific, Waltham) adjusted to conditions of 5% CO_2_ at 37 °C.

### Cell transfection

The siRNAs targeting mTOR were designed by the Genepharma (Shanghai, China). The oligonucleotides sequences of the siRNAs were as follows: mTOR siRNA-1 5′- GGCCAUAGCUAGCCUCAUATT-3′; mTOR siRNA-2 5′- GAGCCUUGUUGAUCCUUAATT-3′; mTOR siRNA-3 5′- GCCCCUACAUGGAGCCUAUTT-3′. The KYSE30 and KYSE150 cells were transfected with the siRNA-mate and a transfection kit (Genepharma, Shanghai, China) following the manufacturer’s guidelines.

### In vitro cytotoxicity

KYSE30, KYSE150, KYSE410 and TE-12 cells were cultured at a density of 5×10^3^ cells /well in 96-well plates. On the other hand, the ESCC cells were incubated with IMMU and IACS for 72 h, and their viability was determined using the Cell Titer-Glo® luminescent cell viability assay kit (G7572, Promega, USA) in line with the manufacturer’s instructions. The luminescence was recorded by SPARK Multiplate Reader (TECAN, Switzerland).

### IncuCyte S3 live cell imaging system assay

ESCC cells were seeded into 96-well plates and cultured for 12 h and treated with IMMU or/and IACS for 72 h. During the culture period, the proliferation of cells was examined, and the plates were placed in the IncuCyte S3 Live Cell Imaging System (Sartorius, Germany) from which the images were acquired every 2 h. Data analysis was performed from the cell confluent degree formation curve using the IncuCyte S3 software.

### ATP assay

Cells were seeded in 6-well plates and incubated with the drugs for 48 h. Then the ATP levels was determined by an ATP Detection Kit (S0027, Beyotime, China) according to the manufacturer’s instructions. The relative light unit (RLU) value was detected using a multimode reader (TECAN, Switzerland).

### Drug combination assay

The ESCC cells were seeded in 96-well plates and incubated with IMMU or/and IACS for 72 h. This was followed by determination of cell viability by the Cell Titer-Glo® luminescent cell viability assay kit (G7572, Promega, USA) using SPARK Multiplate Reader (TECAN, Switzerland). Next, drug synergy scoring was performed on the SynergyFinder website (https://synergyfinder.fimm.fi). The response surface model and zero interaction potency (ZIP) calculation method was employed. ZIP scores ≥ 0 or < 0 indicated a synergistic or antagonistic effect, respectively. ZIP scores > 10 indicated strong synergy.

### EdU staining assay

The Edu staining was performed on ESCC cells seeded in 12-well plates in the presence of IMMU or/and IACS for 48 h. Subsequently, the proliferation of cells was detected using a EdU Cell Proliferation Kit with Alexa Fluor 555 (CX003, epizyme, China) following the manufacturer’s instructions. Images were captured using the laser scanning confocal microscope (STELLARIS 5, Leica, Germany).

### Clonogenic assay

For the clonogenic assay, the KYSE30 and KYSE150 cell lines were seeded in 12-well plates at a density of 500/well. They were then incubated with IMMU, IACS, or combination of IMMU and IACS for 10 days. Next, they were fixed with 4% paraformaldehyde for 30 min at room temperature and stained with 0.01% (w/v) crystal violet for 10 min. The stained plates were imaged, and the total colony area was calculated using the ImageJ software.

### Apoptosis

KYSE30 and KYSE150 cells were incubated in 6-well plates and treated IMMU, IACS or their combination for 48 h. Cell death was detected using an Annexin V-FITC apoptosis kit (Beyotime Biotechnology, China). The stained cells were then analyzed by fluorescence-activated cell sorting (FACS) (FACSCantoII, BD, USA).

### Migration and invasion

Transwell chambers (Corning, New York, USA) were utilized to monitor the perform migration and invasion experiments. The cells were resuspended in a FBS-free starvation medium, after which 2 × 10^3^ cells were seeded into the upper chamber of a 24-well Transwell plate (Corning, New York, USA). To conduct the invasion assay, upper chamber membranes were pre-coated with the Matrigel (Corning, New York, USA) before they were seeded. The cells were incubated with IMMU, IACS, or a combination of both for 48 h, respectively. The cells were fixed with 4% paraformaldehyde for 30 min and then stained with 0.01% (w/v) crystal violet for 15 min at room temperature. Finally, the images were analyzed using Image J software.

### Reactive oxygen species assay

The ESCC cells were inoculated into 6-well plates and incubated with the drugs for 48 h. Total reactive oxygen species (ROS) level was quantified using the ROS Assay Kit (S0033, Beyotime, China) following the manufacturer’s instructions. Finally, the samples loaded with the probe were detected using the FACS (BD, FACSCantoII, USA) or examined using an fluorescence microscope (Leica, Germany).

### Caspase-3 activity and mitochondrial membrane potential

To evaluate caspase-3 activity and mitochondrial membrane potential, the Caspase-3 Activity and Mitochondrial Membrane Potential Assay Kit (C1073M, Beyotime, China) was employed according to the manufacturer’s protocol. Green fluorescence from the GreenNuc™ DNA-binding complex indicated caspase-3 activation, while mitochondrial membrane potential was assessed by the accumulation of Mito-Tracker Deep Red 633, which emits deep red fluorescence in a potential-dependent manner. Next, the cell culture medium was aspirated, washed once with PBS, mixed with 300 μL of the detection buffer and then incubated at room temperature in the dark for 30 min. Finally, it was observed using a fluorescence microscope (Leica, Germany).

### RNA-Seq analysis

KYSE30 cells were incubated with the 6 μg/mL IMMU, 6 μM IACS, or their combination for 24 h. Total RNA was extracted with TRIzol (TransGen Biotech). RNA-sequencing analysis was conducted by the Novogene (Tianjin, China). To perform RNA-seq analysis, DEGs were selected based on the following threshold: Padj ≤ 0.05 and |log_2_fold change | ≥ 0.26. Differential gene enrichment analyses were subjected to the ‌Gene Ontology (GO) and Kyoto Encyclopedia of Genes and Genomes (KEGG) analyses. Moreover, the gene set enrichment analysis (GSEA) was conducted using the Molecular Signatures Database (MSigDB) hallmark gene set and Go gene set. The GSEA Analysis tool is available at http://www.broadinsti tute.org/gsea/index.jsp.

### Immunofluorescence

Briefly, the ESCC cells were seeded directly onto coverslips in 12-well plates and allowed to adhere overnight. The cells were then fixed with 4% paraformaldehyde (PFA) at room temperature for 30 min and treated with 0.1% Tween-20 for 10 min. They were washed and blocked with 5% bovine serum albumin (BSA) for 1 h and incubated with primary antibodies at 4 °C overnight. The next day, the cells were incubated with a fluorescent secondary antibody for 1 h at room temperature. Nuclear staining was performed by addition of the DAPI reagent (epizyme). Images were captured using a laser scanning confocal microscope (STELLARIS 5, Leica, Germany).

### Immunohistochemistry and Hematoxylin-eosin assays

Paraffin embedded tissues from 222 ESCC patients were obtained from the Department of Pathology at the First Affiliated Hospital of Gannan Medical University in line with the Declaration of Helsinki guidelines. The samples were examined by specialists following the World Health Organization standards. The use of the specimens and associated patient data was authorized by the Ethics Committee of the First Affiliated Hospital, Gannan Medical University. ESCC PDXOs and PDXs were fixed with formalin and embedded in paraffin. Hematoxylin-eosin (H&E) staining was performed using a standard histological method. Next, IHC staining was carried out using the UltraSensitive^TM^ SP (mouse/rabbit) kit, IHC kit, and DAB kit (MXB, Fuzhou, China) following standard procedures. The Panoramic Histiocyte Quantitative Analysis System (TissueFAXS Plus, TissueGnostics, Austria) was employed to capture images.

### Western blot

Cell lysates were subjected to the SDS lysis buffer (Beyotime Biotechnology, Shanghai, China) enriched with the PMSF (Solarbio, Beijing, China) and Phosphatase Inhibitor Cocktail (Beyotime Biotechnology, Shanghai, China). The proteins were separated through electrophoresis using SDS-PAGE gel and then transferred to PVDF membranes (Millipore, Darmstadt, Germany). The membrane was incubated with 5% skim milk to block nonspecific binding. They were subsequently incubated with primary antibodies overnight at 4 °C, followed by HRP-conjugated secondary antibodies specific to the primary antibodies at room temperature (Supplementary Table 1). Finally, protein bands were visualized using the ECL reagent (Cytiva, USA) and the signals were captured using a Bio-Rad Multifunctional chemiluminescence imaging system.

### ESCC organoids generation and real-time organoid viability analysis

The ESCC organoids extracted from the ESCC PDX xenograft tissues were prepared using ESCC organoids kits (bioGenous, Hangzhou, China) in line with standard procedure. 50 µL of suspension in Matrigel was added into each well of a 24-well plate and incubated for 10 min at 37 °C for polymerization following the addition of 500 µL complete Medium to each well. The organoids were cultured in a humidified incubator with 5% CO_2_ at 37 °C.

To test the viability of organoids, the ESCC organoids were seeded in 96-well plates and treated with IMMU, IACS, or their combination for 6 days. The IncuCyte S3 live cell analysis system (Sartorius, Gorteen, Germany) was then utilized to analyze the viability of the cells.

### Animal experiments

Fresh tumor tissues were obtained from the First Affiliated Hospital of Gannan Medical University, Ganzhou, China, from 2022 to 2024. This study was approved by the Clinical Research Ethics Committee of the First Affiliated Hospital of Gannan Medical University. The procedures for the collection and use of tissues were performed in accordance with the guidelines of the Helsinki Declaration of 2013 (no. 2016-16). Informed consent was obtained from all patients.

The NOD/SCID female mice, aged 5 to 6 weeks, were purchased from the Huachuang Sino Inc (Taizhou, China). The study protocol was approved by the Biomedical Research Ethics Committee, Gannan Medical University, Jiangxi, China on 18 March 2022 (No. 2022301), and performed in full compliance with these guidelines. Briefly, the ESCC xenograft tumor tissues were cut into pieces of 2 to 3 mm^3^ and implanted into the right flank of mice. The tumors were allowed to grow and when the average tumor volume reached approximately 100–150mm^3^, the tumor-bearing mice were randomly assigned to 4 groups (Vehicle, 10 mg/kg IMMU, 2.5 mg/kg IACS, and their combination). IMMU was intravenously injected one day a week for three weeks, The IACS was intragastrically administrated five days a week for 4 weeks. At 29 days after treatment, the mice were euthanized through euthanasia. The tumor size and body weight of the mice were measured twice a week using a caliper, and the tumor volume was calculated using the formula: tumor volume (mm^3^) = length × (width)^2^ × 0.5. The tumor growth inhibition (TGI) rate was calculated as follows: (1-treated tumor volume/control tumor volume) ×100%. At the end of the experiment, mice were euthanized if tumor volumes did not exceed 2000 mm³. All ESCC PDX xenograft tumors and major organs were harvested for subsequent molecular and pathological analyses. To assess liver and kidney function, serum levels of alanine aminotransferase (ALT), aspartate aminotransferase (AST), blood urea nitrogen (BUN), and creatinine (Cr) were measured using commercial assay kits (Jiancheng Bio, Nanjing, China).

### Statistical analysis

All experimental data are presented as mean values ± standard error of the mean (SEM) using GraphPad Prism 9 software. The IC50 values were calculated through nonlinear regression analysis of the concentration-response curves in SPSS 16.0. Significance was calculated by two-tailed unpaired Student’s t test, without adjustment or one-way analysis of variance (ANOVA), with Dunnett’s multiple comparisons test, with the untreated or vehicle control group as the reference. For all experiments, results with *P* < 0.05 were considered significant and are denoted in the Figs. ns indicates a non-significant difference. *p < 0.05; **p < 0.01; ***p < 0.001.

## Results

### Profiling the expression of TROP2 in ESCC

**Fig. 1:**
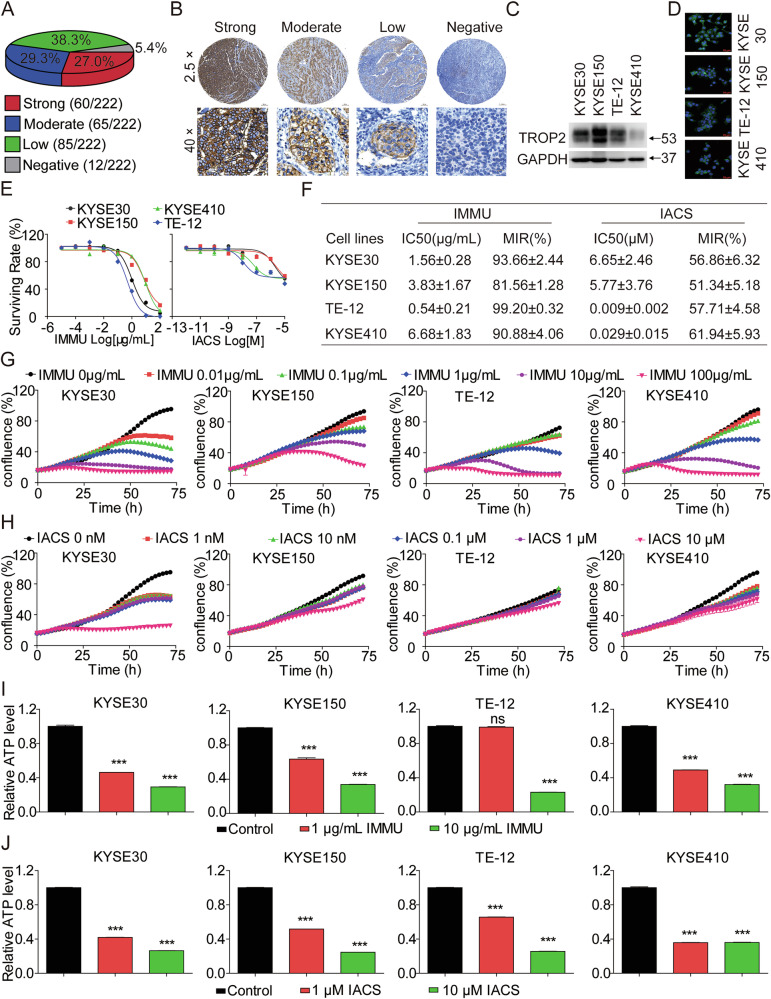
Antiproliferative effects of IMMU and IACS alone on TROP2-positive ESCC in vitro*.* **A** The percentage of TROP2 expression in 222 ESCC tumors, classified into four staining intensities as follows: strong, moderate, low, and negative. **B** Representative images showing the 4 different staining intensities for TROP2 as determined by the IHC assay on human ESCC tumor tissue microarrays (TMA). Images were captured at ×25 magnification (upper panels) and ×40 magnification (lower panels), with scale bars = 200 µm (upper panels) and scale bars = 20 µm (lower panels), respectively. **C**, **D** Analysis of the expression levels of TROP2 in ESCC cell lines as determined by western blotting and IF assay. Representative IF images captured at ×400 magnification, with scale bars = 20 µm. **E**, **F** KYSE30, KYSE150, TE-12, and KYSE410 cells were treated with IMMU or IACS for 72 h, respectively. The viability of cells in the indicated groups was measured using the Cell Titer-Glo cytotoxicity assays. **G**, **H** ESCC cells exposed to IMMU or IACS from 0 to 72 h were examined using the Incucyte S3 real-time cell analysis system. **I**, **J** The cellular ATP production level of ESCC cells incubated with IMMU or IACS alone was measured using the ATP Detection Kit. Data are shown as the mean ± standard error of the mean (SEM) of at least three independent experiments, and statistical significance was determined using unpaired T test (ns, not significant; *p < 0.05; **p < 0.01; ***p < 0.001).

To investigate the expression profile of TROP2 in ESCC, we performed IHC on tissue microarrays comprising 222 cores of human ESCC specimens. It was observed that TROP2 was overexpressed in ESCC specimens. Specifically, TROP2 tested positive in 94.6% (210/222) of the 222 ESCC patient specimens. Particularly, TROP2 exhibited negative, low, moderate, and high staining intensities in 5.4% (12/222), 38.3% (85/222), 29.3% (65/222) and 27.0% (60/222) of specimens, respectively (Fig. 1A, B). To identified the ESCC cell lines for subsequent in vitro evaluation, we quantified the expression level of TROP2 in 4 ESCC cell lines through western blotting and immunofluorescence. There results indicated that TROP2 was overexpressed in KYSE30, KYSE150, TE-12, and KYSE410 cell lines (Fig. 1C, D). Therefore, the 4 human ESCC cell lines were selected for experiments to test the in vitro efficacy of IMMU (FDA-approved ADC targeted TROP2) and IACS (OXPHOS inhibitor).

### IMMU and IACS alone remarkably inhibited the proliferation of ESCC cells in vitro

Initially, we explored the in vitro cytotoxicity of IMMU and IACS in KYSE30, KYSE150, TE-12, and KYSE410 cells through the Cell Titer-Glo Cell Viability assay. The analysis revealed that IMMU significantly inhibited the growth of the four ESCC cell lines in a dose-dependent manner. Specifically, the IC50 values of KYSE30, KYSE150, TE-12 and KYSE410 cells were 1.56 ± 0.28 μg/mL (MIR = 93.66 ± 2.44%), 3.83 ± 1.67 μg/mL (MIR = 81.56 ± 1.28%), 0.54 ± 0.21 μg/mL (MIR = 99.20 ± 0.32%), and 6.68 ± 1.83 μg/mL (MIR = 90.88 ± 4.06%), respectively. Furthermore, the IACS exerted moderate inhibitory effects in KYSE30, KYSE150, TE-12 and KYSE410 cells, with IC50 values of 6.65 ± 2.46 μM (MIR = 56.86 ± 6.32%), 5.77 ± 3.76 μM (MIR = 51.34 ± 5.18%), 0.009 ± 0.002 μM (MIR = 57.71 ± 4.58%), and 0.029 ± 0.015 μM (MIR = 61.94 ± 5.93%), respectively (Fig. 1E, F).

Subsequently, we examined the individual effects of IMMU and IACS on KYSE30, KYSE150, TE-12, and KYSE410 cells using the IncuCyte® S3 live-cell imaging system, a non-invasive, long-term, and real-time dynamic imaging system for quantitative analysis of live cells. This test indicated that IMMU suppressed the growth of ESCC cells in a dose- and time-dependent manner. Similarly, IACS dose- and time-dependently inhibited ESCC cells, KYSE30, KYSE150, and KYSE410, but this effect was observed in TE-12 (Fig. 1G, H, Supplementary Fig. 1). Moreover, we validated the effects of IMMU and IACS alone on the mitochondrial ATP production in ESCC cells. The analysis confirmed that IMMU and IACS dose-dependently inhibited ATP level in KYSE30, KYSE150, TE-12, and KYSE410 cells (Fig. 1I, J).

Collectively, the present results demonstrated that IMMU alone achieved strong tumor inhibitory effect in vitro, but IACS alone only displayed moderate antitumoral activity in vitro.

### IMMU and IACS exerts synergistic effects on ESCC cells in vitro

Combinational therapy is commonly used in biomedical research and clinical applications. In recent years, there has been enhanced focus on exploring the therapeutic efficacy of combination of ADCs with other anticancer medications [34, 35]. Here, we investigated whether the combinational of IMMU and IACS can treat ESCC.

**Fig. 2:**
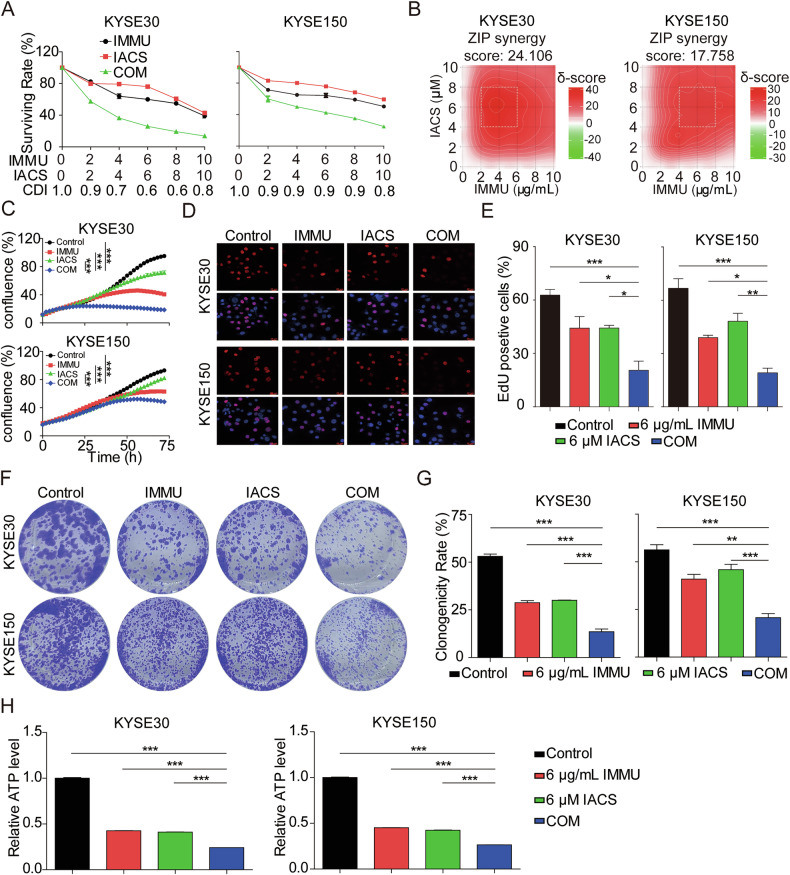
Synergistic antitumoral effects on ESCC in vitro by IMMU plus IACS. **A** KYSE30 and KYSE150 cells incubated with various concentrations of IMMU, IACS or their combination (COM) for 72 h. The cells were subjected to Cell Titer-Glo cytotoxicity assays. A CDI value< or>1 indicates a synergistic or antagonistic effect. **B** Heatmaps showing the responses to drug combination of KYSE30 and KYSE150 cells. ZIP Synergy scores were calculated using Synergyfinder software. Scores > 0 indicates synergism, and scores >10 were considered strong synergistic. **C** Detection of KYSE30 and KYSE150 cells treated with IMMU, IACS, or their COM for different times using Incucyte S3 real-time cell analysis system. **D**, **E** Results of the EdU assays indicating the proliferation of the KYSE30 and KYSE150 cells treated with IMMU, IACS or their COM for 48 h. Images were captured using a laser scanning confocal microscope and shown at ×400 magnification, with scale bars = 20 µm. **F**, **G** The antiproliferative activity was measured by assessing the area of colonies stained with crystal violet. **H** The cellular ATP production level of KYSE30 and KYSE150 was detected using an ATP Detection Kit. Data are shown as the mean ± standard error of the mean (SEM) of at least three independent experiments, and statistical significance was determined by unpaired T test (ns, not significant; *p < 0.05; **p < 0.01; ***p < 0.001).

Initially, we tested the impact of these drugs on the viability of KYSE30 and KYSE150 cells via the Cell TiterGlo Cell Viability assay. IMMU was applied at concentrations of 0, 2, 4, 6, 8, and 10 μg/mL, while IACS was administered at concentrations of 0, 2, 4, 6, 8 and 10 μM. The results uncovered that the two drugs showed synergistic effects in all combined doses (CDI value < 1). The combination of 6 μg/mL IMMU and 6 μM IACS had the most significant pronounced effects, with CDI values of 0.6 and 0.9 for KYSE30 and KYSE150 cells, respectively (Fig. 2A). Using the revised concentration gradients and inhibition indices, drug synergy was evaluated with the ZIP model via the SynergyFinder online platform [36]. The analysis revealed average synergistic anti-tumor responses of 24.11% in KYSE30 cells and 17.76% in KYSE150 cells. Similarly, the combination of IMMU and IACS exhibited strong synergistic effects on antitumoral activity (ZIP synergy scores>10). The white rectangle shows the area of the maximum synergistic area, and the result indicated that the concentration of 6 μg/mL IMMU and 6 μM IACS was the encompassing the region of highest synergy (Fig. 2B). Therefore, we selected 6 μg/mL IMMU and 6 μM IACS as the optimal concentration for subsequent in vitro experiments.

In addition, we explored the synergistic effect of 6 μg/mL IMMU combined with 6 μM IACS in KYSE30, KYSE150 cells using IncuCyte® S3 live-cell imaging system, EDU assay, colony formation, and ATP Assay. It was observed that IMMU combined with IACS synergistically and time-dependently inhibited the growth more strongly compared to IMMU or IACS alone (Fig. 2C, Supplementary Fig. 2). Furthermore, EdU staining and colony formation assays revealed that the combination of IMMU and IACS significantly suppressed the proliferation of KYSE30 and KYSE150 cells compared to monotherapy groups (Fig. 2D–G). Furthermore, we found that IMMU combined with IACS synergistically decreased ATP levels in KYSE30 and KYSE150 cells relative to monotherapy groups (Fig. 2H).

Collectively, IMMU combined with IACS synergizes to suppress the ESCC cell growth in vitro.

### IMMU synergizes with IACS to induce mitochondrial apoptosis, migration and invasion

To clarify the mechanisms mediating the antitumoral activity of IMMU combined with IACS in vitro, we analyzed apoptosis, oxidative stress, and migration and invasion processes.

**Fig. 3:**
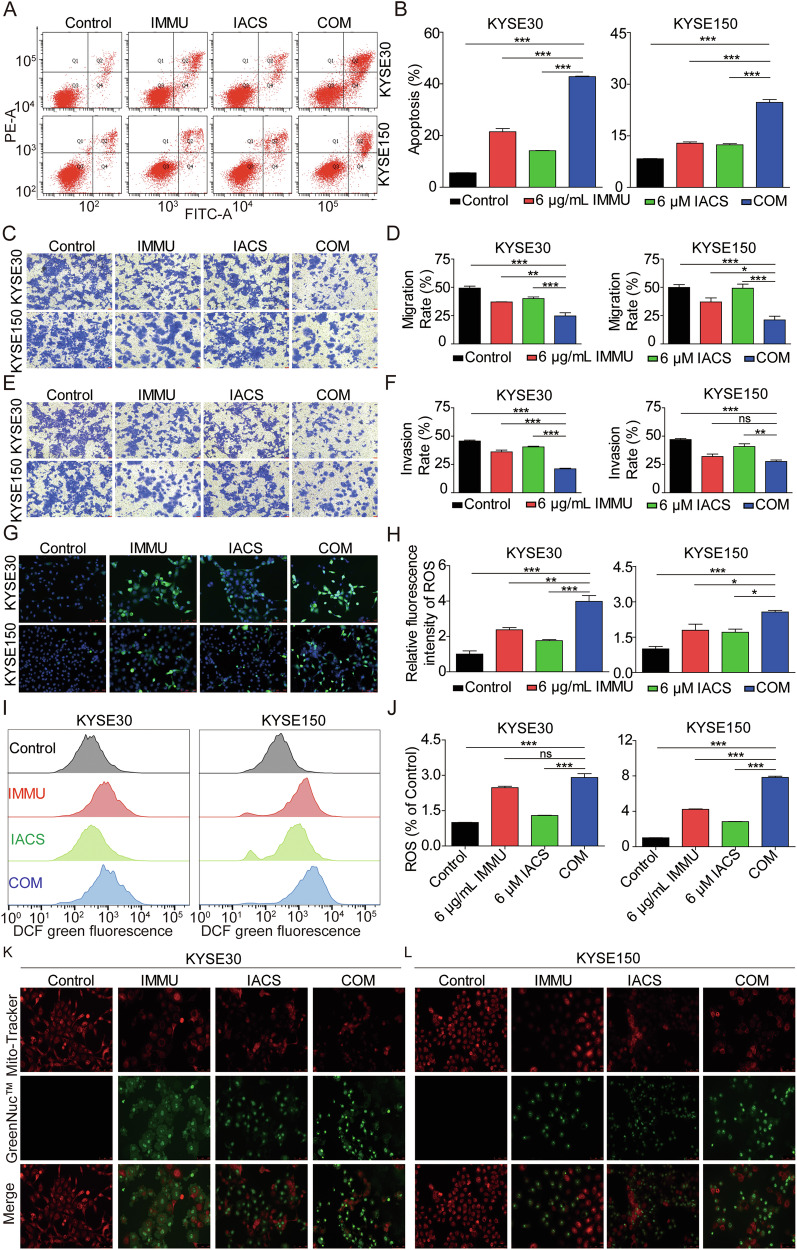
IMMU combined with IACS synergistically induced mitochondrial apoptosis and inhibited cell motility in ESCC cells. KYSE30 and KYSE150 cells were treated with IMMU, IACS, or their COM for 48 h. **A**, **B** Results of the FACS showing the degree of apoptosis. Migration (**C**, **D**) and invasion (**E**, **F**) of IMMU, IACS, or their COM treatment cells as determined by the Transwell assays. Scale bars, 250 μm. **G**, **H** ROS production was estimated using DCFH-DA kits. ROS levels were quantified using Image J software. ROS marker DCF is shown in green. Scale bars, 100 μm. **I**, **J** ROS levels were quantitatively analyzed using FACS. Representative FACS histograms showing ROS levels in the indicated groups. **K**, **L** Caspase-3 Activity and Mitochondrial Membrane Potential assay. Red represents Mitochondrial membrane potential signal; green represents Caspase-3 enzyme activity. Scale bars, 100 μm. Data are shown as the mean ± SEM of triplicate measurements. Groups were compared using one-way ANOVA. ns (not significant), *p < 0.05; **p < 0.01; ***p < 0.001.

First, we explored whether the combination of IMMU and IACS influenced cell apoptosis. Results of the Annexin V-FITC staining based on FACS revealed that IMMU and IACS alone moderately increased apoptotic cells. However, the combination treatment markedly increased the number of apoptotic cells relative to the monotherapy groups (Fig. 3A, B). Furthermore, we explored the impact of the combination treatment on the motility of KYSE30 and KYSE150 cells through transwell cell migration/invasion assays. The results indicated that the combination treatment synergistically inhibited the migration and invasion of the two cells lines more strongly compared monotherapy groups (Fig. 3C–F).

Moreover, we tested whether the combination treatment increased oxidative stress in ESCC cells. Analysis of the results indicate that the combination treatment synergistically increased the level of ROS in both cell lines compared to monotherapy groups (Fig. 3G–J), suggesting that IMMU combined with IACS induces cellular oxidative stress. This indicates that the combination of IMMU and IACS can synergistically promote cell apoptosis and oxidative stress.

To confirm clarify the role of mitochondria in the occurrence of cell apoptosis following exposure to the combination therapy, we measured the Caspase-3 activity and mitochondrial membrane potential. Notably, IMMU or IACS applied separately induced apoptosis and increased Caspase-3 enzyme activity (green light), and decreased the mitochondrial membrane potential decreases (red light). On the other hand, the above effects were more pronounced in the combination therapy group in KYSE30 (Fig. 3K) and KYSE150 (Fig. 3L) cells, respectively. Therefore, the combination therapy impaired the mitochondrial membrane potential and promoted cell apoptosis.

Altogether, these findings demonstrated that the combination of IMMU and IACS synergistically inhibited tumor cell proliferation via inducing apoptosis, oxidative stress, and suppressing the migration and invasion, promoting the ESCC death.

### Combination treatment of IMMU and IACS significantly induced mitochondrial apoptosis via the PI3K-AKT-mTOR pathway

**Fig. 4:**
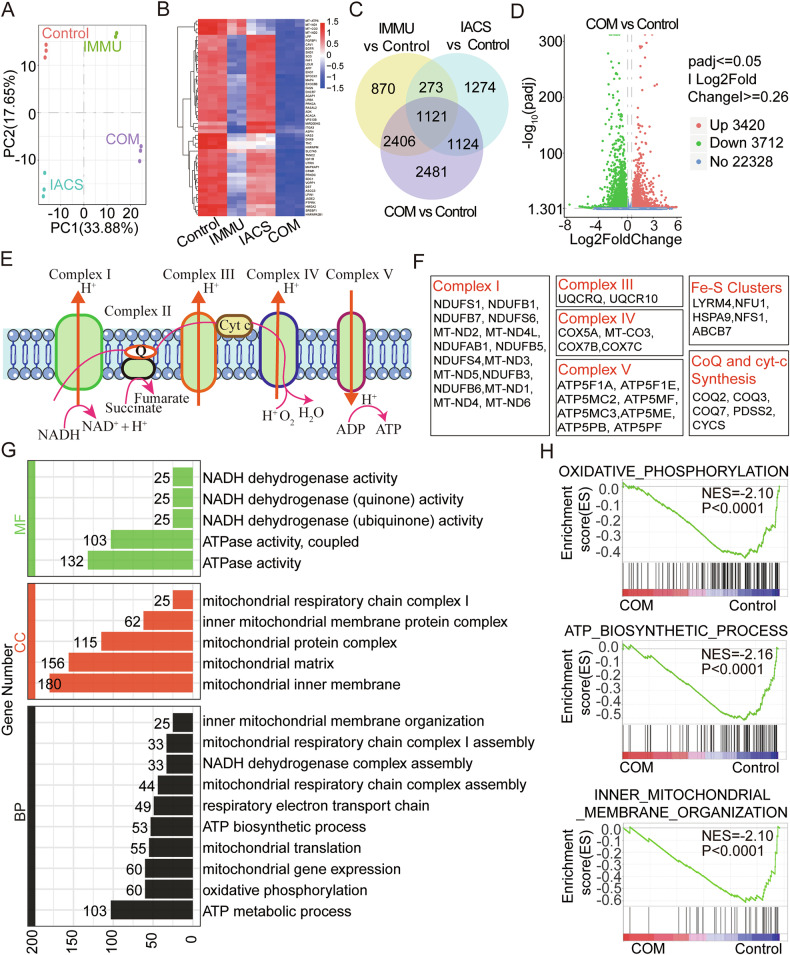
RNA-seq showed cellular OXPHOS-dependence upon combination treatment of IMMU and IACS in ESCC cells. Gene expression tests were conducted using the RNA-seq in KYSE30 cells exposed to 6 μg/mL IMMU, 6 μg/mL IACS, alone or in combination for 24 h. **A** Genetic dissimilarity among the samples as determined by Principal Component Analysis (PCA). **B** Heatmap showing the top 50 significantly downregulated genes in the transcriptomes of KYSE30 cells treated with Control, IMMU, IACS, or COM (*n* = 3). Significant differential expression was defined as an absolute log2 (fold change) ≥ 0.26 and q < 0.05. **C** The Venn diagram showing the number of overlapping DEGs in IMMU, IACS and their COM compared to control group. **D** Volcano plot of the identified DEGs between the control and COM including up-regulated (red) and down-regulated (green) genes in KYSE30 cells. **E**, **F** An illustration of ATP produced by the OXPHOS pathway through the transport of electrons to various transmembrane protein complexes. KEGG analysis demonstrating significant enrichment of the OXPHOS metabolic pathway in the top 3000 negative enrichment genes. **G** GO enrichment analysis showing negative enrichment for OXPHOS and mitochondria respiration pathway. The DEGs were highly enriched in biological processes (BP), cellular components (CC), and molecular function (MF). **H** GSEA of gene sets which were altered in the COM treatment group compared to the Contral group. Enrichment graph of oxidative phosphorylation, ATP biosynthetic process and inner mitochondrial membrane. See also Supplementary Fig. 3.

To clarify the molecular mechanisms mediating the effects of the combination treatment, RNA-seq analysis was performed on the KYSE30 cells exposed to 6 μg/mL IMMU and 6 μM IACS, alone or in combination. Results of the PCA and Heatmap identified a distinct separation in gene expression between samples from the combination group and monotherapy groups (Fig. 4A, B, Supplementary Fig. 3), which demonstrated that IMMU combined with IACS altered the gene expression. The combination treatment induced differential expression of 7132 genes (DEGs) compared to the control group, among which 3420 were upregulated and 3712 were downregulated (Fig. 4C, D). In further experiments, we tested the changes in expression of the downregulated genes following exposure to the combination treatment. Specifically, we conducted KEGG analysis which indicated that the DEGs were associated with OXPHOS. Most of the OXPHOS-associated factors that influenced the sensitivity of cells to the combination treatment were directly involved in the electron transport chain (ETC) complex I (NDUFS1, NDUFB1, NDUFB7, NDUFS6, MT-ND2, MT-ND4L, NDUFAB1, etc.), complex III (UQCRQ, UQCR10), complex IV (COX5A, MT-CO3, COX7B, COX7C), and complex V (ATP5F1A, ATP5F1E, ATP5MC2, ATP5MF, ATР5MC3, ATP5ME, ATP5PB, ATP5PF) (Fig. 4E, F). These factors, which play a role in the OXPHOS complex assembly and mitochondria protein synthesis, may influence response to the combination treatment (Fig. 4E, F). To test the association of the combination treatment with OXPHOS, we performed Gene ontology (GO) analysis of the DEGs. Analysis of the enrichment data showed that the DEGs were associated with the processes of OXPHOS, ATP biosynthetic process, Inner Mitochondrial membrane organization, Mitochondrial respiratory chain complex assembly, Mitochondrial translation and Mitochondrial gene expression (Fig. 4G). Gene set enrichment analysis (GSEA) also indicated marked enrichment in the OXPHOS-related processes, including OXPHOS (NES = –2.10, p < 0.0001), ATP biosynthetic process (NES = -2.16, p < 0.0001), mitochondrial inner membrane (NES = –2.10, p < 0.0001), as well as those associated with mitochondria respiration (Fig. 4H, Supplementary Fig. 4). Collectively, these findings revealed that the genes associated with the OXPHOS may influence the observed synergistic effects.

**Fig. 5:**
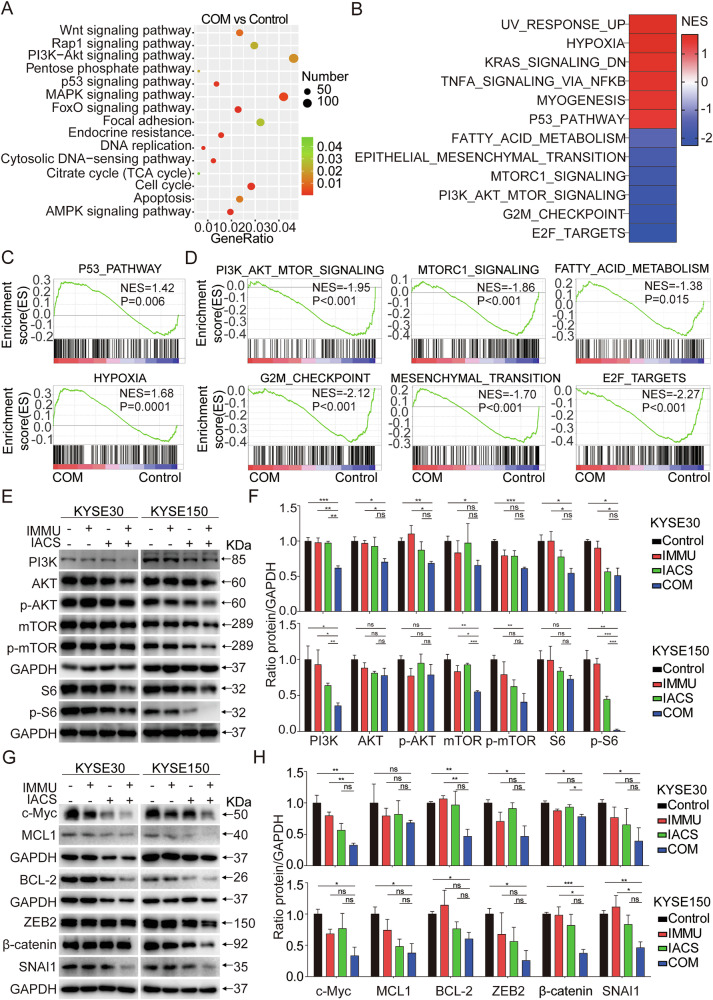
IMMU and IACS combination treatment affected cancer-related signaling pathways. **A** KEGG pathway analysis of DEGs in RNA-seq of KYSE30 cell following COM treatment. **B** Gene Set Enrichment Analysis (GSEA) of KYSE30 cell after COM treatment using HALLMARK gene sets database in MSigDB. **C** GSEA indicating strong positive enrichment of pathways, such as P53 and HYPOXIA after COM treatment. **D** Negative enrichment was observed for the PI3K/AKT/mTOR signaling pathway, mTORC1 signaling pathway, fatty acid metabolism, G2/M checkpoints, mesenchymal transition, and E2F targets. NES Normalized Enrichment Score. **E**–**H** Analysis of the PI3K/AKT/mTOR signaling pathway, apoptosis-related and migration-related proteins in KYSE30 and KYSE150 cells by Western blotting. GAPDH served as the loading control. Data are shown as the mean ± SEM of triplicate measurements. Data were compared by one-way ANOVA. ns (not significant), *p < 0.05; **p < 0.01; ***p < 0.001.

To further dissect the functional significance of these DEGs, KEGG analysis was performed, which revealed that the combination treatment modulated multiple signaling pathways in KYSE30 cells. Specifically, the combination treatment altered pathways, such as PI3K-AKT-mTOR pathway, p53 pathway, MAPK pathway, focal adhesion, FOXO pathway, apoptosis, cell cycle, and AMPK pathway (Fig. 5A). Moreover, GSEA analysis of the common DEGs in KYSE30 cells exposed to the combination treatment revealed strong positive enrichment in cell cycle progression and proliferation, including p53 pathway (p = 0.006; NES = 1.42) and a hallmark_of hyoxia (p < 0.0001; NES = 1.68) (Fig. 5B, C). We also observed strong negative enrichment of gene sets involved in PI3K_AKT_mTOR_SIGNALING (p < 0.001; NES = -1.95), MTORC1_SIGNALING (p < 0.001; NES = -1.86), FATTY_ACID_METABLISM (p = 0.015; NES = –1.38), G2M_CHECKPOINT (p < 0.001; NES = –2.12), MESENCHYMAL_TRANSITION (p < 0.001; NES = -1.70), and E2F_TARGETS (p < 0.001; NES = –2.27) (Fig. 5B, D). These findings provide crucial insights into the biological processes and pathways targeted by the combination treatment. These pathways can modulate the cancer cell proliferation and metastasis. Furthermore, western blot analysis confirmed that the combination treatment downregulated key proteins associated with the AKT-mTOR pathway, apoptosis, and migration and invasion in KYSE30 cells, similar to the findings obtained in the RNA-seq analysis (Fig. 5E–H). These results reveal that the combination treatment can effectively block several cancer-related pathways.

**Fig. 6:**
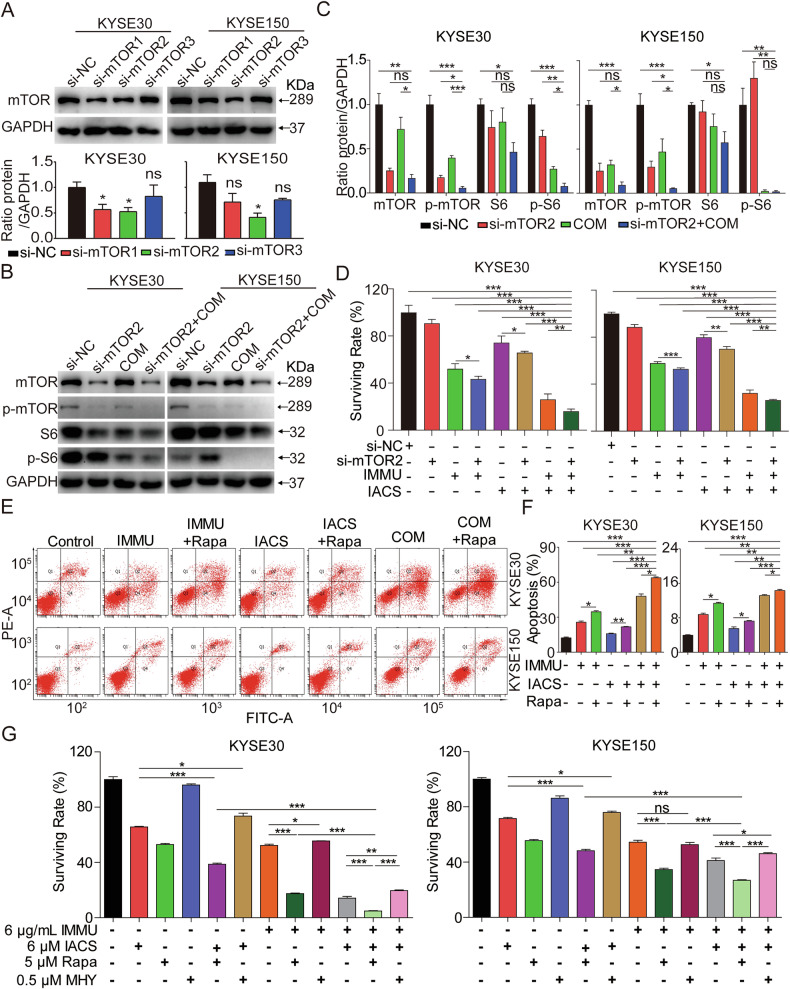
mTOR knockdown inhibited ESCC cell growth and induced apoptosis. **A** mTOR knockdown efficiency in KYSE30 and KYSE150 cells was confirmed via Western blotting. **B**, **C** KYSE30 and KYSE150 cells with mTOR knockdown were incubated with IMMU and IACS for 24 h, and the total and phosphorylated protein levels of mTOR and S6 were determined using Western blotting analysis. **D** The effect of combination therapy on the viability of KYSE30 and KYSE150 cells with mTOR-knockdown was explored using Cell TiterGlo Cell Viability assay. **E**, **F** Apoptosis was analyzed by FACS, with Annexin V/PI-positive cells shown in bar charts. **G** The viability of KYSE30 and KYSE150 cells treated with IMMU (6 μg/mL), IACS (6 μM), Rapamycin (5 μM) or MHY1485 (0.5 μM) alone or in combinational for 72 h. Cell viability was measured using the Cell Titer-Glo cytotoxicity assay. Data are presented as the mean ± SEM. A two-tailed unpaired Student’s ttest was employed for statistical analysis (**A**, **D**, **F**, **G**) or one-way ANOVA (**C**). ns (not significant), *p < 0.05; **p < 0.01; ***p < 0.001.

To clarify the downstream signaling pathways affected by the combination therapy, we generated mTOR-knockdown KYSE30 and KYSE150 cells through small interfering RNA (siRNA). Western blotting analysis revealed that si-mTOM2 efficiently silenced mTOM protein expression in both cell lines (Fig. 6A). It was further observed that mTOR knockdown suppressed the phosphorylation levels of mTOR and S6 in both cell lines (Fig. 6B, C). In addition, we investigated the cell viability following exposure to the combination therapy in mTOR-knockdown KYSE30 and KYSE150 cells using Cell TiterGlo Cell Viability assay. The combination therapy exhibited a stronger inhibitory effect relative to that of other treatment groups in the same cells (Fig. 6D). To determine evaluate the effect of the combination therapy on the mTOR pathway, we assessed the effect of rapamycin, a small molecule mTOR inhibitor. Flow cytometry assays revealed that the combinational therapy significantly increased cell apoptosis compared to the treatment groups in both cell lines (Fig. 6E, F).

Next, the impact of the IMMU alone or in combination with IACS on the AKT-mTOR pathway was tested. The results revealed that 5 μM rapamycin significantly enhanced the anti-proliferative activity of IMMU alone or in combination with IACS in KYSE30 and KYSE150 cells. Specifically, rapamycin combined with IMMU or IACS exhibited more potent inhibitory effects relative to the monotherapy groups. In addition, the combined treatment of rapamycin, IMMU and IACS achieved more significant anti-proliferative activity compared to IMMU combined with IACS in KYSE30 and KYSE150 cells (Fig. 6G). MHY1485, an mTOR activator, was able to reverse the inhibitory effects of IMMU, both alone and when combined with IACS, thereby enhancing cell survival (Fig. 6G). These findings collectively indicate that IMMU, either as a monotherapy or in combination with IACS, suppresses tumor growth in KYSE30 and KYSE150 cells by targeting the AKT-mTOR signaling pathway.

In summary, the present results show that the combination treatment efficiently inhibited several cancer-driving signaling pathways and more broadly affected other pathways. Based on these findings, further investigations are needed to examine the therapeutic potential of these pathways in ESCC.

### Combination treatment of IMMU and IACS affected unique EC-associated prognostic genes

To verify the biological relevance of the genes regulated by the combination treatment, we explored the association of the TOP1000 (500 upregulated and 500 downregulated) DEGs with overall survival through Kaplan-Meier curves in the esophageal cancer cohort from The Cancer Genome Atlas (TCGA) datasets. In the TCGA cohort, 11 genes were strongly associated with overall survival and correlated with gene expression patterns in cancer and normal paracancer tissues. Decreased levels of COL4A1, DEK, GINS1, HPCAL1, KIF4A and RBMX predicted longer survival in esophageal cancer patients (Supplementary Fig. 5A), while overexpression of IGFL1P1, LRRC57A-AS1, SNHG3, ULK3 and ZFYVE19 correlated with longer survival (Supplementary Fig. 5B).

Moreover, the expression levels of these genes in esophageal cancer tissues and in the combined treatment group was analyzed. The results showed that COL4A1, DEK, GINS1, HPCAL1, KIF4A and RBMX were significantly increased in esophageal cancer tissues and decreased in the combined treatment group, suggesting that are potential prognostic markers (Supplementary Fig. 6A). In contrast, IGFL1P1, LRRC57A-AS1, SNHG3, ULK3 and ZFYVE19 showed poor expression in esophageal cancer tissues but high expression in the combined treatment group (Supplementary Fig. 6B).

Overall, these results reveal the function and prognostic value of genes targeted by the combined treatment. These findings indicate that the DEGs participate in esophageal cancer and modulating these genes can potentially improve the overall survival rates of esophageal cancer patients. This discovery is expected to reveal the underlying mechanisms and design drugs targeting this disease.

### IMMU combined with IACS synergistically inhibited PDXOs growth of advanced ESCC

**Fig. 7:**
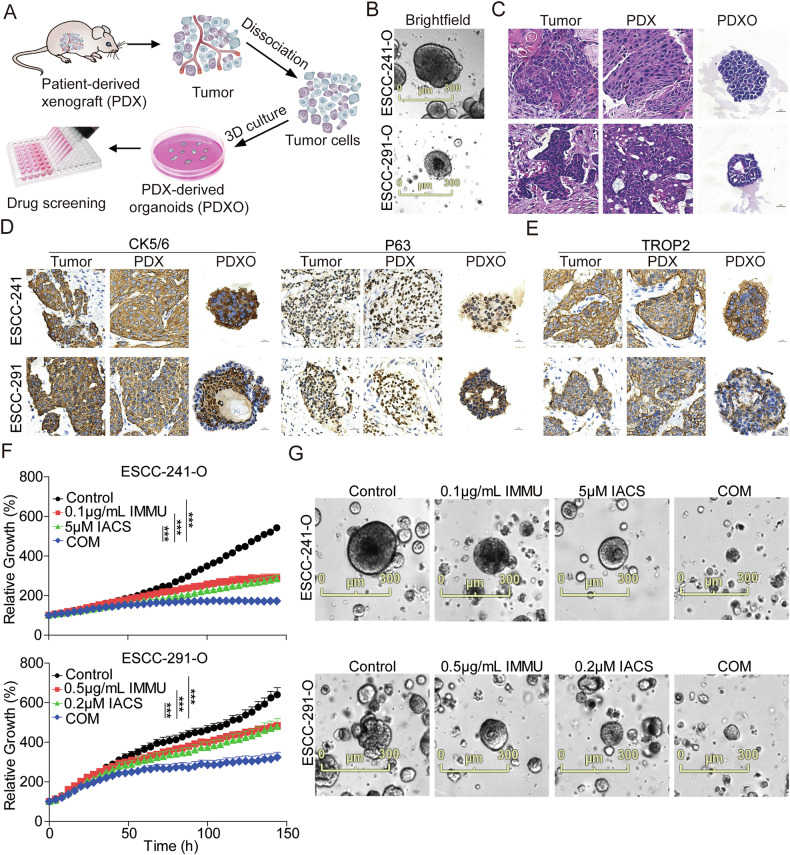
Synergistic antitumoral effects of IMMU combined with IACS on ESCC PDXOs. **A** Schematic diagram illustrating the generation of ESCC PDX-derived organoids (PDXOs) from their corresponding PDX tumors for drug tests. **B** Brightfield pictures of ESCC-241-O and ESCC-291-O models. Scale bar = 300 μm. **C** Representative HE staining images of ESCC primary tumors, PDXs and their corresponding PDXOs. Images were captured at ×400 magnification. Scale bars = 20 µm. Representative IHC staining images of ESCC markers including CK5/6 and P63 (**D**) and TROP2 expression (**E**) in ESCC primary tumors, PDXs and their corresponding PDXOs. DAPI nuclear stain is shown in blue. Images are captured at the ×400 magnification. Scale bars = 20 µm. **F** ESCC-241-O and ESCC-291-O were treated with IMMU and IACS either alone or in combination from 0 h to 144 h. Organoid size (%) was calculated using Incucyte S3 Zoom software from the phase-contrast images. **G** Brightfield images of PDXOs treated with IMMU or IACS either alone or in combination at 144 h. Scale bar = 300 μm. Each data point represents triplicate wells. Data are presented as the mean ± standard error of the mean (SEM) of at least three independent experiments, and groups were compared using the unpaired T test (ns, not significant; *p < 0.05; **p < 0.01; ***p < 0.001).

Patient-derived xenograft (PDX), patient-derived organoid (PDO) or PDX tumor-derived organoid (PDXO) models, which possess the principal pathologic and genetic characteristics of their original tumors, have emerged as an alternative method for modeling patient diseases [37]. In our study, we constructed ESCC PDXO models derived from their corresponding PDX tumors (Fig. 7A). The analysis indicated that the PDXOs exhibited varied growth rates and morphologies (Fig. 7B). To examine whether PDXO models recapitulate the biology and architecture of PDX models, we performed H&E and IHC assays on primary tumors, PDX and PDXO sections. Analysis of the H&E assay showed that the PDXOs retained heterogeneous morphologies seen in primary tumors and PDX tissues, ranging from thin-walled cystic structures to solid/compact structures (Fig. 7C). Further IHC assays showed that PDXOs and their corresponding primary tumors and PDX tissues exhibited similar expression patterns of markers such as CK5/6 and P63 (Fig. 7D). They also revealed that TROP2 was overexpressed and exhibited similar subcellular localization in PDXOs to their corresponding primary tumors and PDX tissues (Fig. 7E). Altogether, these results showed that PDXOs recapitulated the histological features and expression of markers of the primary tumors, which is line with previous reports [38]. We further investigated the synergistic anti-proliferative effects of the combination treatment in ESCC-241 and ESCC-291 PDXOs using IncuCyte® S3 live-cell imaging system. Results of the live cell imaging analysis demonstrated that the combination treatment synergistically and time-dependently inhibited the growth more strongly compared to monotherapy groups from 0 to 144 h in the two PDXOs (Fig. 7F, G).

Based on these findings, we infer that the combination treatment synergistically exerted anti-tumor effects in PDXOs, achieving higher therapeutic efficacy ex vivo, and can be used to guide the selection of PDX models in vivo.

### Combination of IMMU and IACS cooperatively improved the anti-tumor activity in multiple advanced ESCC PDXs

**Fig. 8:**
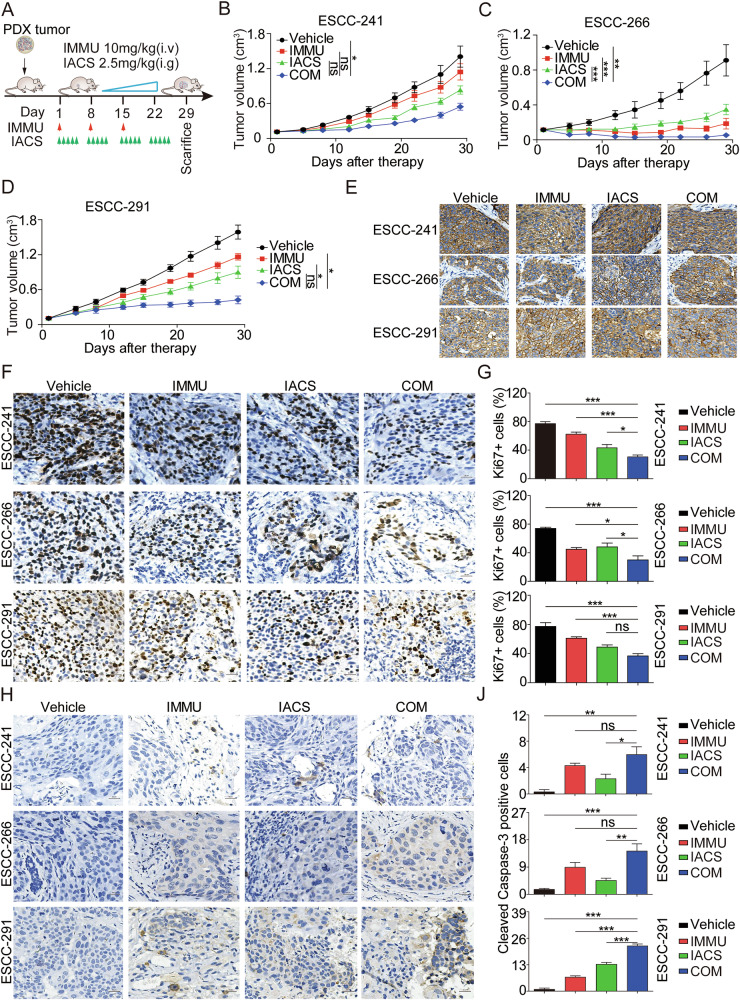
Combination treatment of IMMU and IACS inhibits ESCC tumor growth in PDX model. **A** Schematic diagram showing the experimental designs. Treatment groups, doses, and schedules are indicated. IMMU (red line, 10 mg/kg) were intravenously administered once a week for 3 times, and IACS (green line, 2.5 mg/kg) was intragastrically administered five times a week for four weeks. Synergistic antitumoral effects of IMMU and IACS on tumor growth in ESCC241 (*n* = 5, **B**), ESCC266 (*n* = 4, **C**) and ESCC291 (*n* = 5, **D**) PDX models. **E** The expression level of TROP2 in tumors from the indicated four groups after specific treatments. Representative images showing the TROP2 expression through IHC examination. **F**–**J** The IHC staining of Ki-67 and cleaved caspase 3 in paraffin-embedded tumor tissue samples from the ESCC241, ESCC266 and ESCC291 PDX models. Images were recorded at ×400 magnification. Scale bars = 20 µm. Data are shown as the mean ± standard error of the mean (SEM) of at least three independent experiments, and statistical significance for various groups was determined by unpaired T test (ns, not significant; *p < 0.05; **p < 0.01; ***p < 0.001).

Next, we further investigated the therapeutic efficacy of the combination treatment of in three PDX models. Therapy was initiated when the average volume of subcutaneous tumors was approximately 150 mm^3^. Tumor-bearing mice were randomly divided into four groups (*n* = 4–5) as follows: vehicle, 10 mg/kg IMMU, 2.5 mg/kg IACS, COM (10 mg/kg IMMU plus 2.5 mg/kg IACS). The experiment was terminated on day 29 following drug administration (Fig. 8A). In the ESCC-241 PDX model, IMMU or IACS as single agents exerted moderate antitumoral effects, and the tumor growth inhibitory rate (TGI) on day 29 was 18.71% for IMMU alone and 40.87% for IACS alone. The combination treatment achieved a better therapeutic effect exceeding that of IMMU and IACS alone, causing a TGI of 61.29%, but the difference was not markedly significant (Fig. 8B). In the ESCC-266 PDX model, treatment with either IMMU or IACS significantly exerted antitumor effects as confirmed by a decrease in tumor volume relative to the vehicle group (*P* < 0.01). The TGIs for IMMU and IACS were 79.62% and 61.77%, respectively. Notably, the combination treatment markedly induced synergistic tumoricidal activity, demonstrating a TGI of 94.20% compared to all other monotherapy groups (Fig. 8C). In the ESCC-291 PDX model, treatment with IMMU or IACS alone induced a moderate decrease in tumor volume, achieving the TGIs of 26.70% and 43.42%, respectively. Notably, the combination treatment demonstrated a markedly superior therapeutic effect compared to IMMU alone and the vehicle control, achieving a TGI rate of 73.37% (Fig. 8D). In summary, these in vivo results underscore the strong antitumor efficacy of the combination therapy across all three TROP2-positive ESCC PDX models.

Subsequently, the ESCC PDX xenograft tumors were harvested for molecular and pathological analysis to corroborate the findings in vitro functional experiments and to test the therapeutic efficacy. The IHC results revealed that the treatments did not alter the expression of TROP2 in three ESCC PDX models (Fig. 8E), demonstrating that IMMU exerted antitumor effects in a TROP2-dependent matter. In addition, the combination treatment reduced the expression level of Ki67 (Fig. 8F, G) and upregulated that of cleaved caspase 3 (Fig. 8H, J) compared to the monotherapy groups in the ESCC PDX tumor tissues.

In addition, the combination treatments were well-tolerated in the ESCC PDX models, causing no significant changes in body weight throughout the experimental period (Supplementary Fig. 7). Additionally, there were no marked toxic side effects on the heart, liver, spleen, lung, and kidney in any treatment group relative to the control (Supplementary Fig. 8). Furthermore, we tested the side effects of the combination therapy by measuring serum alanine amino transferase (ALT) and aspartate aminotransferase (AST) as the index for liver function, and serum creatinine (Crea) and blood urea nitrogen (BUN) as the index for renal function. We observed that the serum levels of ALT, AST, Crea, and BUN were within the normal range post-treatment, suggesting that the treatment did not induce toxic effects on the liver and kidney models (Supplemental Fig. 9). This further revealed that this combination therapy exerted minimal in vivo toxicity.

Altogether, these results demonstrate that the combination treatment of IMMU and IACS synergistically exerts good therapeutic efficacy in vivo. This provides a strong rationale for the subsequent clinical use of the combination treatments for the treatment of patients with ESCC.

## Discussion

Esophageal cancer is the eighth most prevalent cancer and the sixth leading cause of cancer-related deaths globally [1]. ESCC, a type of esophageal cancer, exhibits a higher incidence compared to the EAC type [39]. Although targeted therapies have been developed, the clinical outcomes of ESCC are still poor. Specifically, there are no reports on the use of TROP2-ADCs and OXPHOS inhibitors to treat advanced ESCC. Here, we investigated the therapeutic effect of IMMU, an FDA-approved ADC targeting TROP2, either alone or in combination with IACS, an oral and selective OXPHOS inhibitor, in advanced ESCC. Our findings demonstrate that TROP2 is expressed in ESCC and that the combination treatment of IMMU and IACS, exerts significant synergistic tumoricidal effects.

TROP2 was highly expressed in tumor tissues compared to normal tissues [40]. Previously, it was reported that TROP2 is predominantly overexpressed in various tumor types, including UC, BC, cervical cancer, CRC, lung cancer, ovarian cancer, pancreatic cancer, thyroid cancer, endometrial cancer [12]. For instance, Hoppe et al. found that TROP2 was expressed in nearly 90% of EAC [24]. Similarly, Wang et al. confirmed the expression of TROP2 in ESCC [41]. Consistently, we uncovered that TROP2 was highly expressed (94.6%) in a ESCC cohort of 222 patients with a majority of cases (56.3%) presenting with moderate to high TROP2 expression. This demonstrates that TROP2 can be a potential target for the treatment of ESCC. Evidence from clinical trials has shown that IMMU has beneficial effects, leading to its FDA approval for the treatment of unresectable locally advanced or metastatic TNBC, unresectable locally advanced or metastatic HR-positive/HER2-negative BC, and locally advanced or metastatic UC. More than 40 clinical trials are underway or scheduled to clarify the effectiveness of IMMU alone or in combination with other therapies for the management of various solid tumors [15]. However, no clinical data have been published. So far, 19 patients with esophageal cancer have been evaluated for the efficacy of IMMU in the IMMU132-01 basket trial, which included a total of 495 patients across various tumor types. Within the esophageal cancer cohort, IMMU demonstrated a relatively low response rate [23]. This may be attributed to prior treatment with irinotecan or to the lack of TROP2 expression. Decrease ADC target antigen expression may simultaneously affect antibody binding, intracellular uptake, and drug release [42–44]. In the IMMU Phase III validation trial ASCENT, the objective response rates of mTNBC patients with high/medium TROP2 expression (44% and 39%) were significantly higher compared with those with low expression (22%) [45, 46]. Elsewhere, in the ASCENT trial, only 1 out of 7 patients with complete TROP2 deficiency exhibited a reduced response [46]. Tumor cells show TROP2 deficiency or gene mutation, making IMMU unable to recognize the abnormal Trop2. Patients with TROP2 gene amplification may achieve a better response to IMMU, however, they might develop TROP2 mutations afterwards, inducing IMMU resistance. In addition, TROP2 mutations (such as T256R) can trigger abnormal protein localization and prevent the binding to IMMU, which limits the drug entry into cancer cells [47]. This suggests the presence of significant heterogeneity in TROP2 expression across different patients and tumors, which in turn influences the treatment outcomes. In future, additional investigations focusing on the TROP2 expression status are advocated needed.

OXPHOS is an energy source which undergoes reprogramming in tumor cells to influence the proliferation of tumor cells, and is considered a therapeutic target for various diseases [48]. Other investigations indicate that overexpression of the OXPHOS gene is negatively correlated with patient survival. In AML, lung cancer, breast cancer, ovarian cancer and other cancers, overexpression of OXPHOS-related genes and increased mitochondrial respiration were observed, indicating that OXPHOS participates in the occurrence of tumor drug resistance and maintenance of tumor cell survival [49]. In AML, the complex I inhibitor IACS significantly inhibits AML cell growth in preclinical models, particularly those sensitive to IDH1/2 mutant AML, with partial remission shown in phase I clinical trials [33, 50]. Studies have reported that IACS yields good therapeutic effects in NSCLC with KRAS mutation or chemotherapy resistance models [51]. In melanoma, IACS inhibited the growth of various melanoma stem cells [52]. In breast cancer, atoquinone inhibits mitochondrial complex III by blocking the HER2/β-catenin signaling pathway, influencing the development of chemotherapy resistance in breast cancer [53]. In ovarian cancer, inhibition of OXPHOS was reported to re-sensitize cisplatin-resistant ovarian cancer cells [54]. In summary, OXPHOS inhibitors, applied as monotherapy or combinations, inhibit energy supply to tumor cells by targeting the mitochondrial energy metabolism pathway, suggesting that it carries therapeutic potential in tumors that rely on OXPHOS.

The ADC combination therapy is often explored in conjunction with chemotherapy, targeted therapy, and immune checkpoint inhibitors [55]. Thus, clinical trials exploring the use of ADCs with OXPHOS inhibitors are limited. For instance, ongoing trials are examining the efficacy of IMMU with ipilimumab+ nivolumab in patients with metastatic UC (NCT04863885) [56, 57]. Moreover, clinical studies are testing the effectiveness of IMMU in combination with DNA damage and repair inhibitors (NCT03992131). Early clinical trial data suggest that the combination of IMMU with immunotherapy or small molecule inhibitors demonstrate good synergistic effects in solid tumors [12, 58]. Moreover, Liu et al. demonstrated that the combination of IACS and venetoclax in AML was more effective compared with single application of either agent [59]. However, given the heterogeneity and individual specificity of tumors, it is clinically challenging to determine the most effective combination therapies. In this study, we systematically investigated the combined effects of IMMU and IACS on ESCC. Results showed that the co-treatment of IMMU and IACS strongly decreased ATP levels and the proliferation of ESSC cells in vitro. Further ex vivo and in vivo experiments revealed that the synergistic effects were also enhanced in PDXO and PDX models treated with IMMU combined with IACS, as compared to either single agent treatment.

To clarify the mechanisms underlying the synergistic effects of IMMU and IACS on ESCC, we tested their impact on apoptosis, oxidative stress, and migration/invasion. This combination therapy suppressed the proliferation of tumor cells by enhancing cell apoptosis, upregulated oxidative stress, and suppressing migration and invasion, all of which promoted ESCC cell death. Apoptosis plays a central role in the maintenance of homeostasis in organisms, and ROS, such as oxygen free radicals, hydroxyl groups, peroxide groups, alkoxy groups, and non-oxygen free radicals, can cause oxidative stress reactions, inducing mitochondrial DNA damage and apoptosis [60, 61]. Data from multiple investigations show that oxidative stress is associated with apoptosis [60, 62]. RNA-seq data also revealed the ESCC cells were functionally dependent on OXPHOS and ROS, suggesting that this combination treatment regulated the glycometabolism in ESCC cells. Impaired mitochondrial OXPHOS is common in cancer cells, although the molecular mechanisms are not well understood [63]. Additionally, OXPHOS inhibition might synergize with other cancer-related pathways to induce ESCC death. KEEG and GSEA data confirmed that the PI3K-AKT-mTOR pathway, focal adhesion, apoptosis, cell cycle, p53 pathway, MAPK pathway, and AMPK pathway, etc. mediated the synergistic effects of IMMU combined with IACS on ESCC. Similar to the RNA-seq analysis results, the combination of IMMU and IACS suppressed the expression levels of p-AKT and p-mTOR. Similarly, the levels of apoptosis-related proteins and epithelial-to-mesenchymal transition (EMT) markers were downregulated in ESCC cells. These data indicated that IMMU, either alone or in combination with IACS, suppressed the tumor growth by downregulating the PI3K-AKT-mTOR signaling pathway in ESCC cells. Hyperactivation of PI3K-AKT-mTOR signaling was implicated in ECSS initiation and/or progression, metastasis and drug resistance [64, 65]. Data show that the PI3K-AKT-mTOR axis was involved in the control of mitochondrial metabolism, TCA cycle and OXPHOS [66, 67]. These results showed that inhibition of PI3K-AKT-mTOR signaling pathway in ESCC cells exposed to the combination of IMMU and IACS decreased mitochondrial respiratory capacity. However, the mechanism by which the combination treatment affects glycometabolism to influence tumourigenesis in ESCC cells need to be further explored.

In summary, we confirm the effectiveness of the combination therapy of IMMU and IACS in multiple esophageal cancer organoids and PDX models, providing a promising treatment strategy for ESCC. However, further study needs to be conducted to fully address clinical transforming potential of the combination therapy. Especially, clinical safety risks such as bone marrow suppression needs to be fully evaluated. Moreover, preclinical models are unable to simulate the ADCC effect of ADC (such as NK cell-mediated killing) due to the lack of functional immune cells, while this mechanism may contribute to partial efficacy in clinical trials. Given the limited numbers of our preclinical models, how well our data can extent to ESCC population heterogeneity needs to be further investigated. Notably, current clinical studies has indicated that at least some patients with low TROP2 expression still responding and those with high TROP2 expression showing no benefit, highlighting the complex relationship between TROP2 expression and ADC efficacy.

In future, we will aim to elucidate the preclinical safety and pharmacokinetic properties of the combination of IMMU and IACS. Identifying and validating gene sets linked to a favorable prognosis (predictive biomarkers) in ESCC patients is an important research focus, which may contribute to the advancement of precision medicine. The strategy of dual targeting TROP2-ADC and OXPHOS inhibitors can adopted to treat other cancers, besides ESCC, and it also includes AML, NSCLC, breast cancer, ovarian cancer, melanoma, etc. Therefore, this study provides an important foundation for future research into the therapeutic efficacy of TROP2 and OXPHOS combination in in other cancers.

## Conclusions

Our data presented herein demonstrate that combination treatment of IMMU and IACS significantly synergizes in suppressing the growth of advanced and TROP2-expressing ESCC. Of greater significance, investigating combined therapies that target dysregulated metabolic pathways like OXPHOS, alongside ADC drugs, represents a promising strategy for treating ESCC and other cancers.

## Supplementary information

SUPPLEMENTAL MATERIAL

SUPPLEMENTAL MATERIAL

## Data Availability

Data are available on reasonable request. The data used to support the findings of this study are available from the corresponding author on request.
