# Supplementary material for: A TROP2-targeting ADC synergizes with oxidative phosphorylation inhibitor to enhance apoptosis in ESCC by suppressing the PI3K-AKT-mTOR signaling pathway
Source: Cell Death Dis. 2025 Dec 1;17(1):67. doi: 10.1038/s41419-025-08278-5 (PMC12827259; doi:10.1038/s41419-025-08278-5)
Supplement: Supplementary file 1 — SUPPLEMENTAL MATERIAL [file 41419_2025_8278_MOESM1_ESM.docx]

# Supplemental information

**Supplemental Table 1. List of antibodies used for immunofluorescence, immunohistochemistry and western blot analysis.**

| Target | Company | Cat No. | Application | Dilution |
| --- | --- | --- | --- | --- |
| TROP2 | Abcam | AB214488 | IF | 1：200 |
| TROP2 | Abcam | AB214488 | WB/IHC | 1：2000 |
| PI3 Kinase p85 Alpha | Proteintech | 60225-1-Ig | WB | 1：1000 |
| AKT | CST | 9272 | WB | 1：2000 |
| Phospho-Akt (Ser473) | CST | 4060 | WB | 1：2000 |
| mTOR | CST | 2983 | WB | 1：2000 |
| Phospho-mTOR (Ser2448) | CST | 2971 | WB | 1：2000 |
| c-MYC | Proteintech | 10828-1-AP | WB | 1：2000 |
| S6 Ribosomal | CST | 2217 | WB | 1：2000 |
| Phospho-S6 Ribosomal | CST | 4858 | WB | 1：2000 |
| MCL1 | Proteintech | 16225-1-AP | WB | 1：2000 |
| Bcl-2 | Affinity | AF6139 | WB | 1：2000 |
| ZEB2 | Proteintech | 14026-1-AP | WB | 1：2000 |
| β-Catenin | Proteintech | 51067-2-AP | WB | 1：2000 |
| SNAI1 | Proteintech | 13099-1-AP | WB | 1：2000 |
| GAPDH | CST | 5174 | WB | 1：10000 |
| Ki-67 (D2H10) Rabbit mAb (IHC Specific) | CST | 9027 | IHC | 1：500 |
| Cleaved Caspase-3 | CST | 9661 | IHC | 1：400 |
| HRP-conjugated Affinipure Goat Anti-Rabbit IgG (H+L) | Proteintech | SA00001-2 | WB | 1：10000 |
| CK5/6 | ZSGB-BIO | ZA-0683 | IHC | 1：100 |
| P63 | ZSGB-BIO | ZM-0406 | IHC | 1：100 |


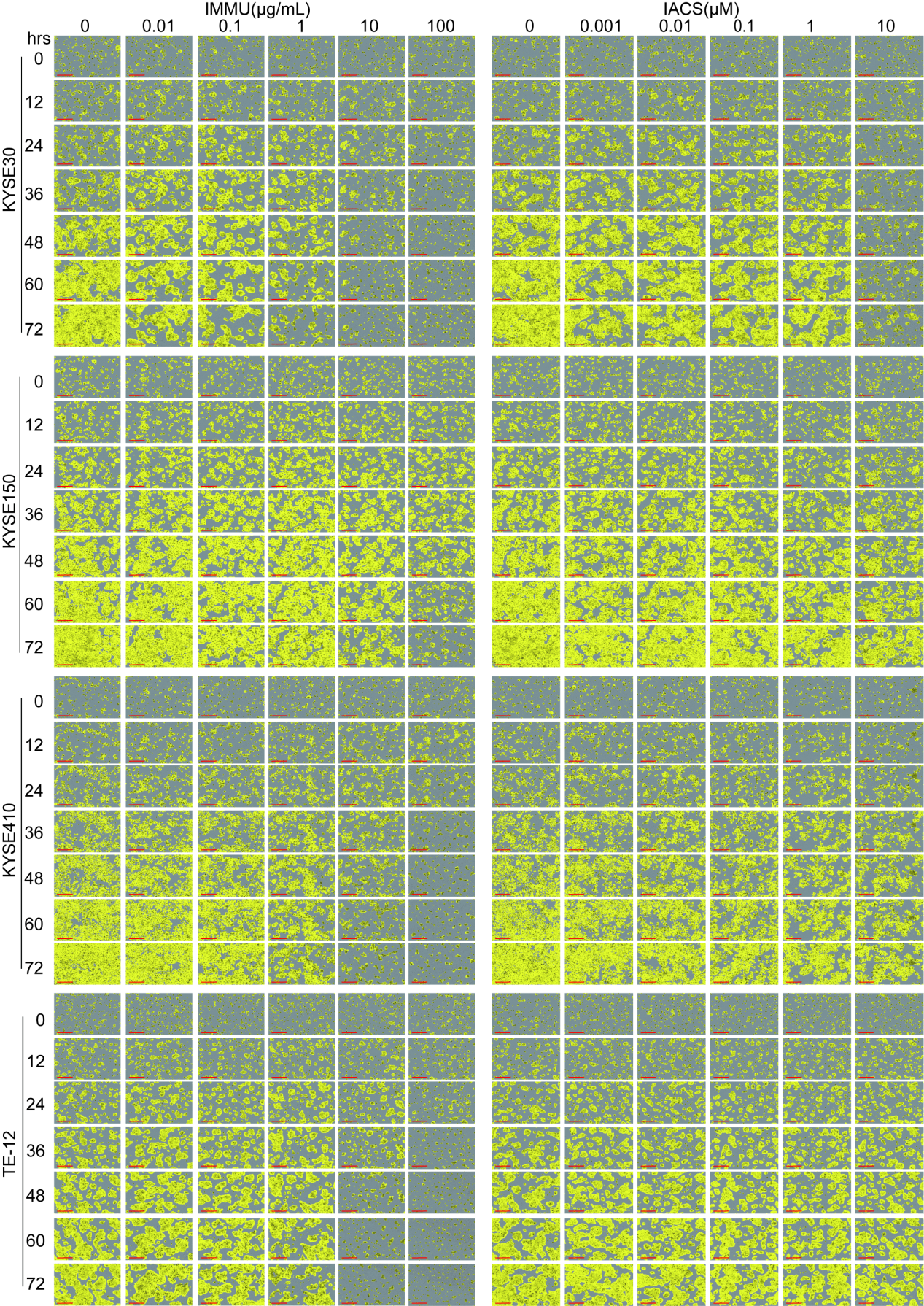


**Supplemental Figure 1. Inhibitory effects of IMMU and IMMU alone on ESCC cells.** ESCC cells were continuously treated with the IMMU and IACS for 72 h, respectively. IncuCyte^®^ S3 Live cell analysis system acquired cell images. Images captured at 100 × magnification, respectively. Scale bars = 400 µm.

**
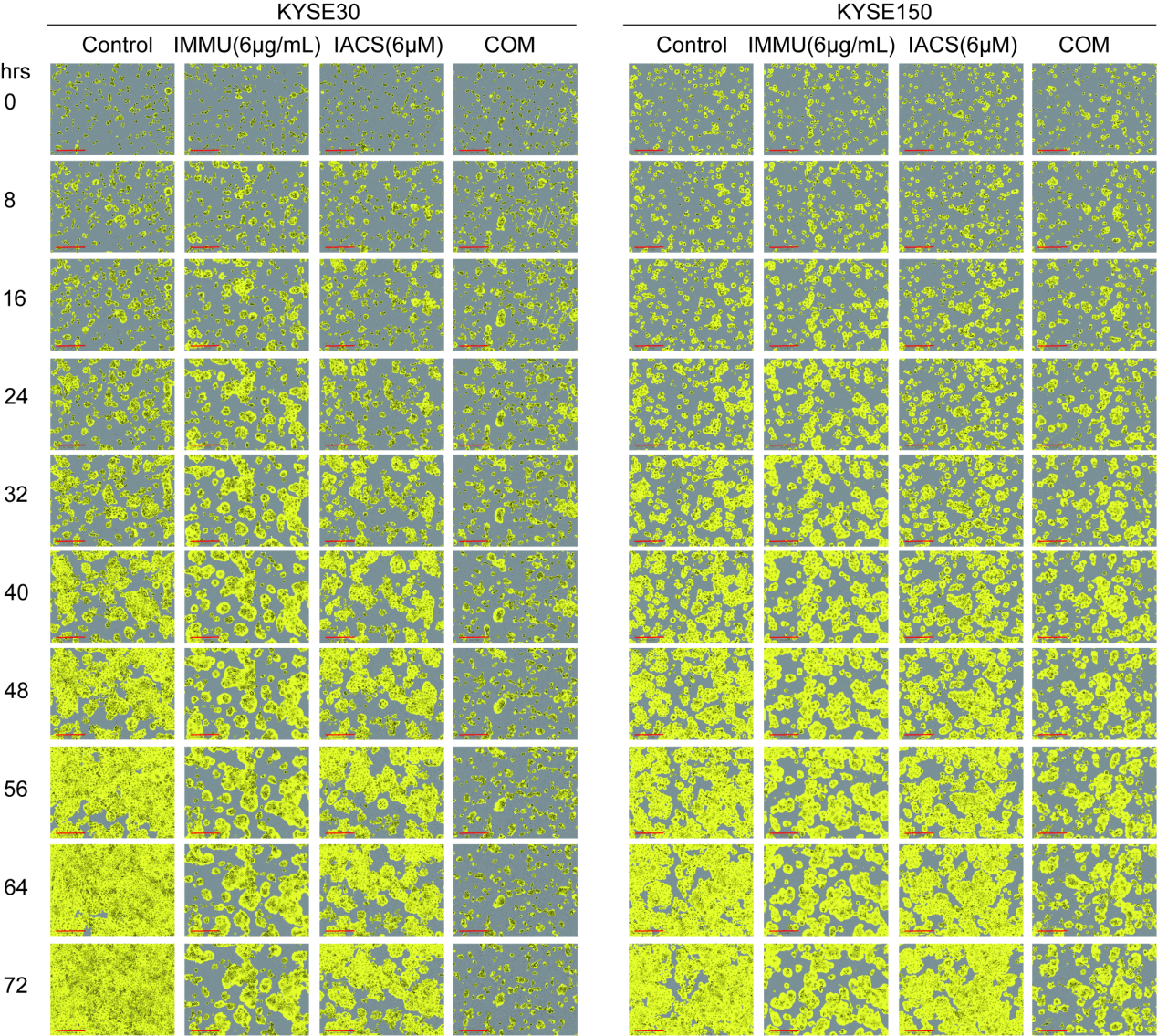
Supplemental Figure 2. Synergistic effects of IMMU plus IACS on ESCC cells *in vitro.*** KYSE30 and KYSE150 cells were treated with IMMU and IACS at different concentration in combination (COM) for 72 h. IncuCyte^®^ S3 Live cell analysis system acquired cell images at the corresponding time points in real time. The pictures of KYSE30 and KYSE150 cells were treated with IMMU and IACS either alone or their COM from 0 h to 72 h. Images captured at 100 × magnification, respectively. Scale bars = 400 µm.

**
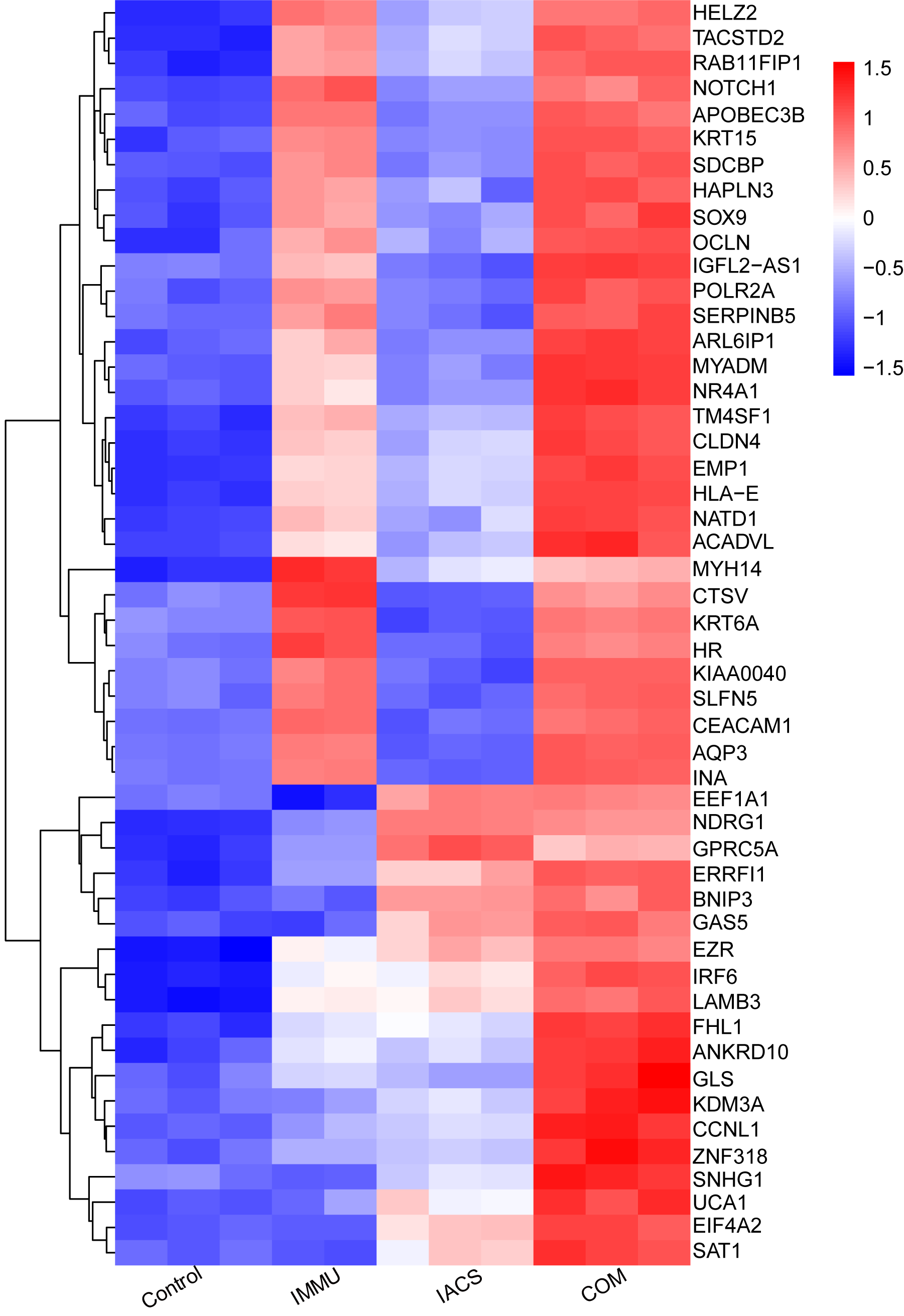
Supplemental Figure 3.** Heatmap showing the top 50 significantly upregulated genes in the transcriptomes of KYSE30 cells treated with Control, IMMU, IMMU, or COM (n = 3). Significant differential expression is defined as an absolute log2 (fold change) ≥ 1 and q < 0.05.


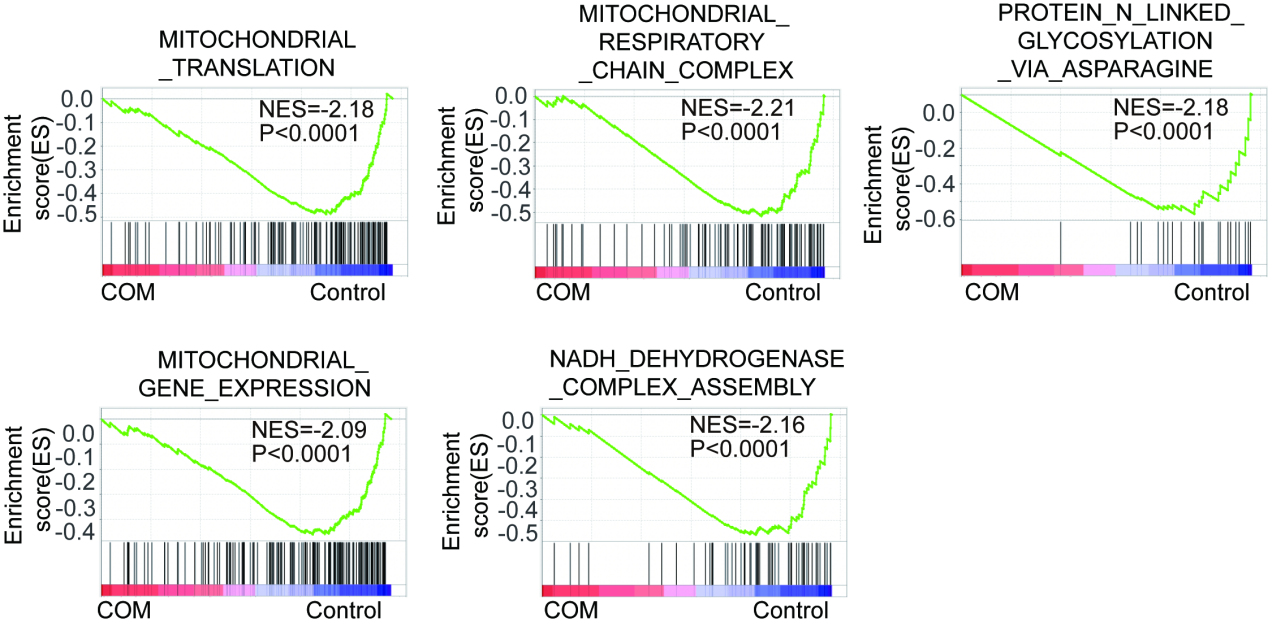


**Supplemental Figure 4.** **RNA-seq revealed cellular OXPHOS-dependence upon combination treatment.** GSEA graphs of top OXPHOS and mitochondria associated gene sets.


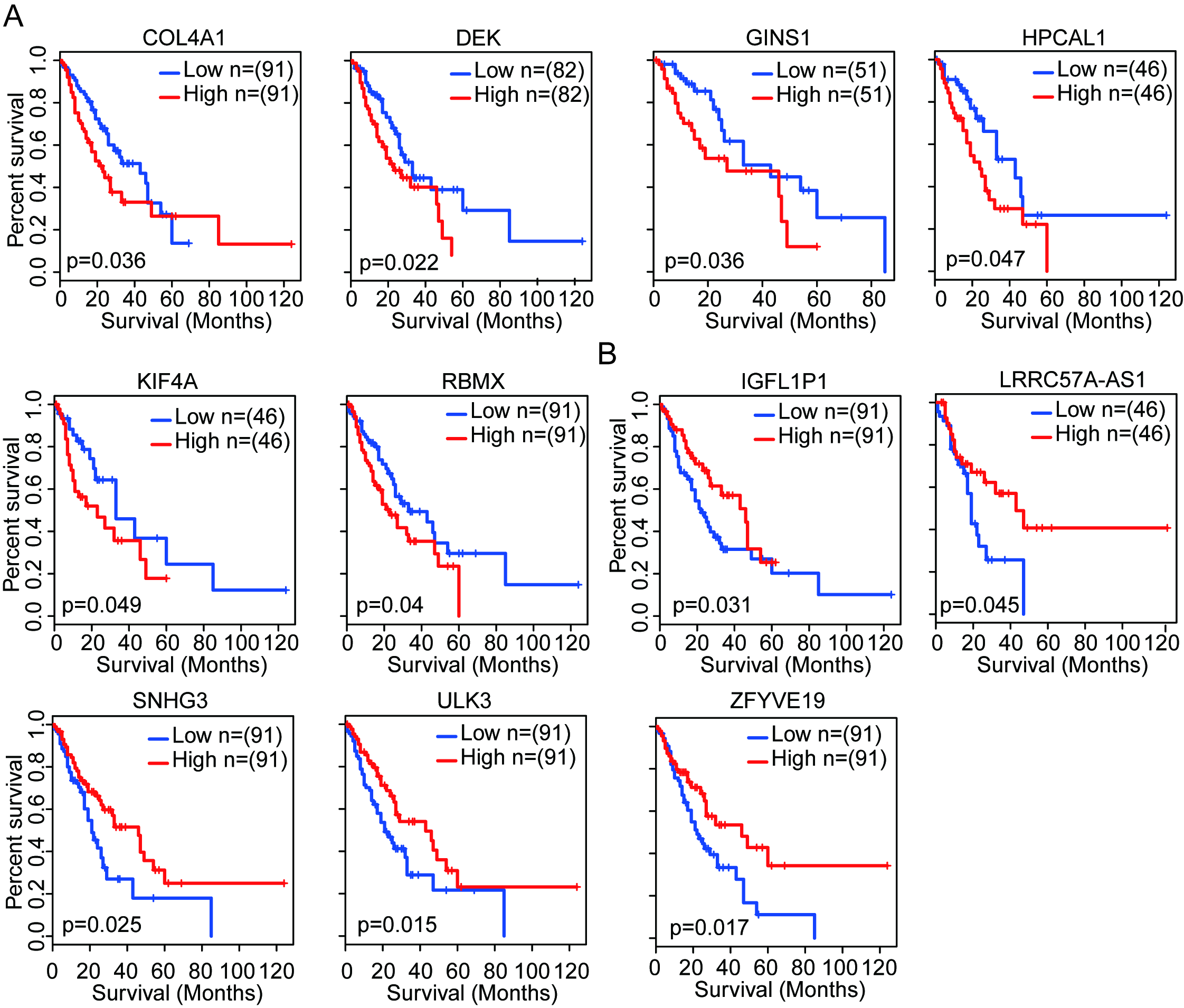


**Supplemental Figure 5. Kaplan-Meier curves regarding 11 survival-related candidate genes.** The relationship between different expressed genes enriched by GSEA and overall survival rate was evaluated using Kaplan-Meier analysis. (A) Lower expression of COL4A1, DEK, GINS1, HPCAL1, KIF4A and RBMX was found to be beneficial to the overall survival rate of patients with ESCC (p<0.01). (B) High expression of IGFL1P1, LRRC57A-AS1, SNHG3, ULK3 and ZFYVE19 significantly improved the overall survival of ESCC patients (p<0.05). Significance was determined using the log-rank (Mantel-Cox) test , and p<0.05 was considered significant.


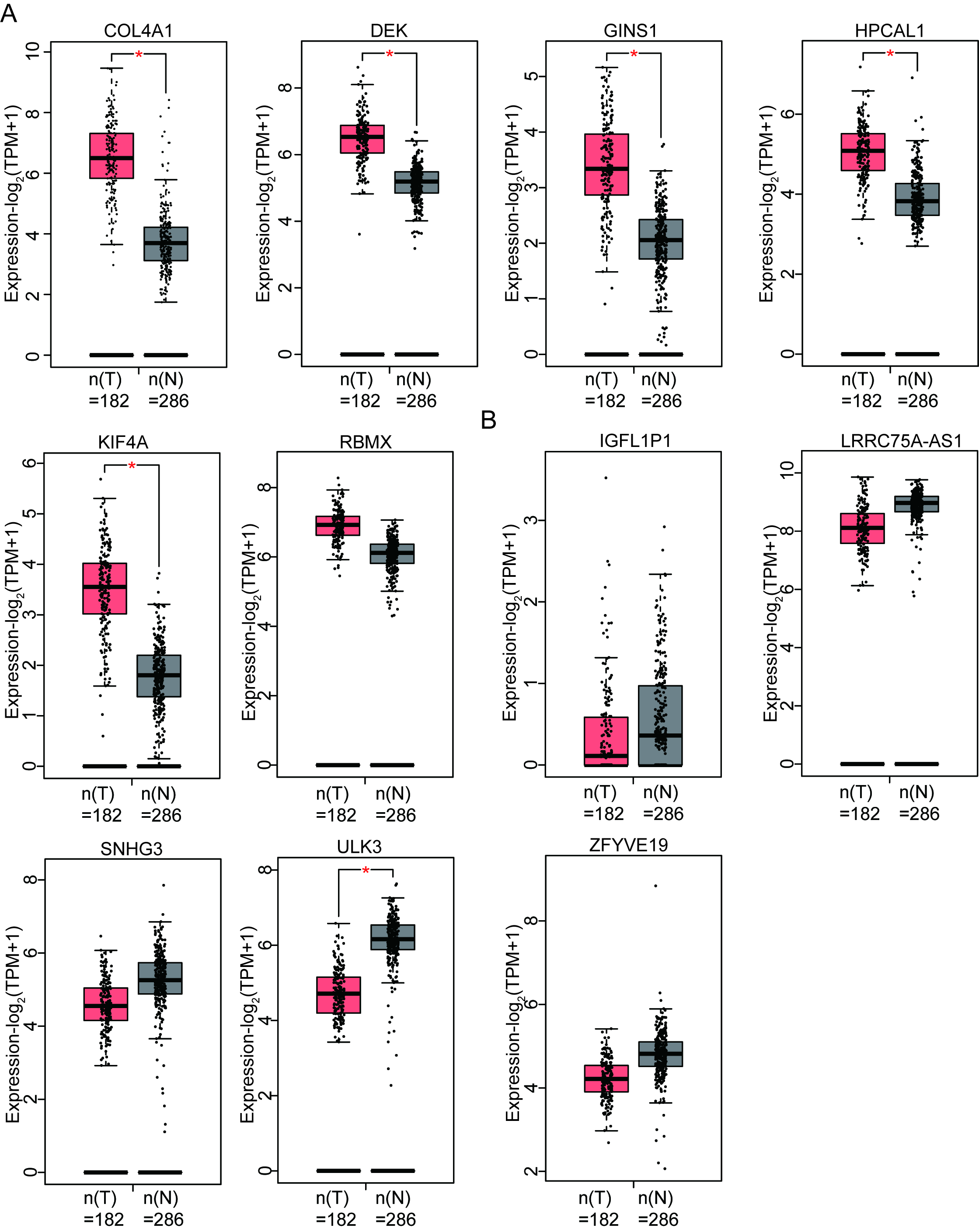


**Supplemental Figure 6. Boxplots regarding the expression pattern of 11 survival-related candidate genes.** (A) The expression of COL4A1, DEK, GINS1, HPCAL1, KIF4A and RBMX genes were high in ESCC in comparison to normal controls. (B) The expression of IGFL1P1, LRRC57A-AS1, SNHG3, ULK3 and ZFYVE19 genes were low in ESCC in comparison to normal controls. p<0.05 is considered significant and was calculated by the two tailed Student’s t test.


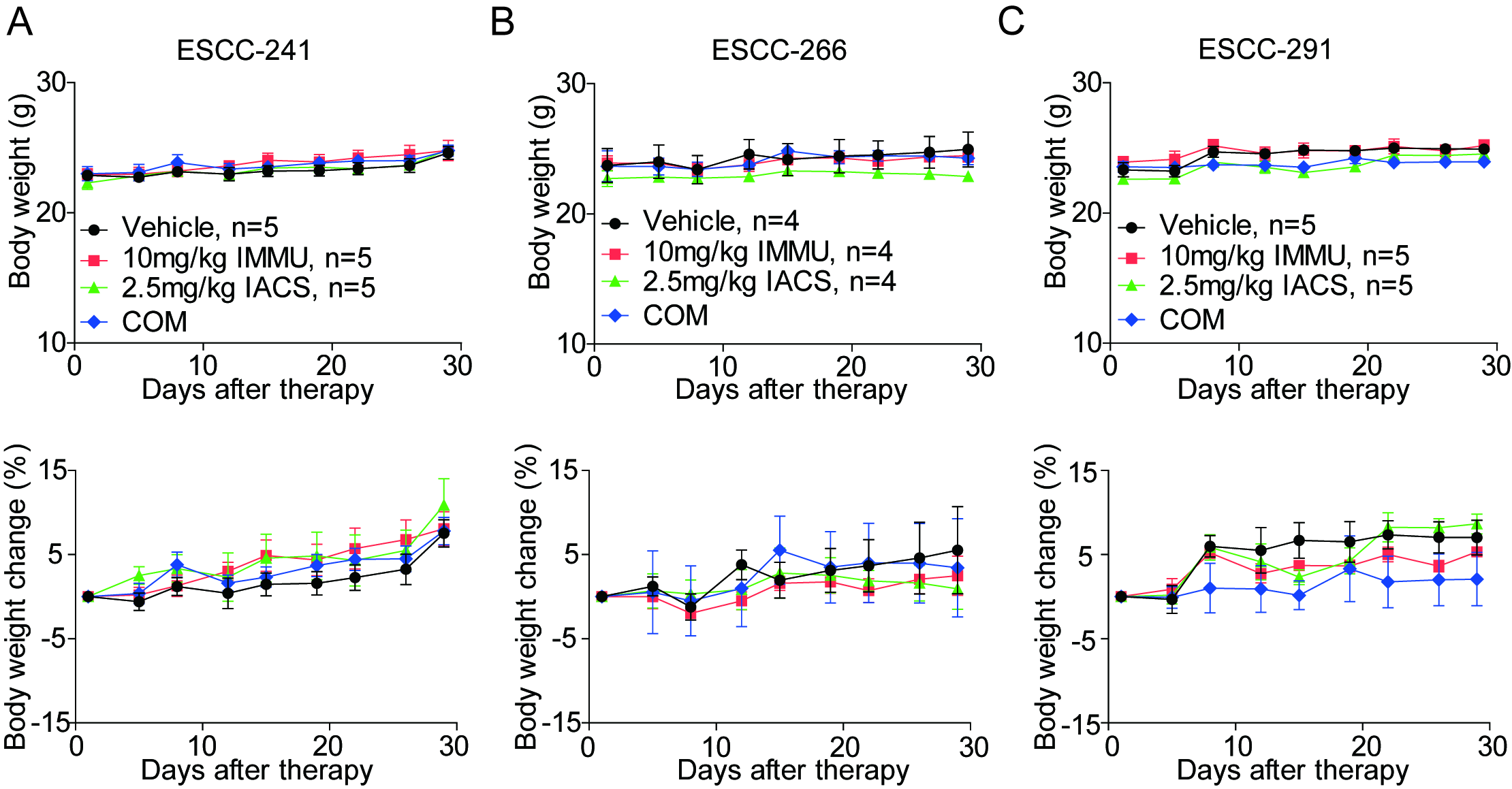


# **Supplementary Figure 7. Synergistic effects of** IMMU and IACS **on body weight of ESCC PDX models.** The weights and their corresponding weight changes of mice following treatment with IMMU and IACS alone or in combination as indicated, were assessed twice every week in ESCC-241 (A), ESCC-266 (B) and ESCC-291 (C) PDX models.


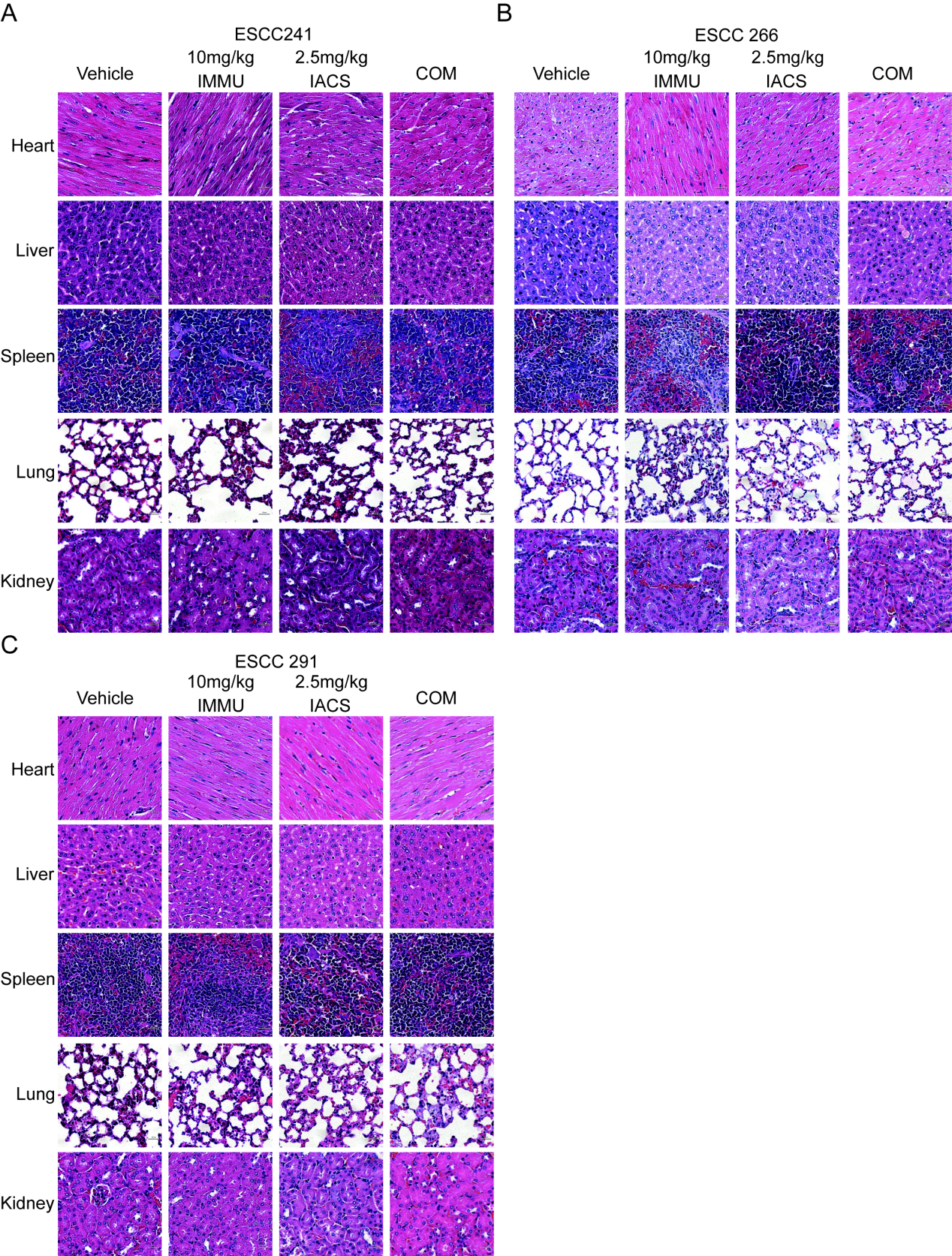


# Supplementary Figure 8. Combined effects of IMMU and IACS on histology of the ESCC PDX models. Treatment of IMMU, IACS, and their COM had no effects on heart, liver, spleen, lung and kidney in ESCC-241 (A), ESCC-266 (B) and ESCC-291 (C) PDX models, proved by H&E assay for the evaluation of pathological changes in these organs of the PDX models. Images were captured at 400 × magnification. Scale bars = 20 µm.


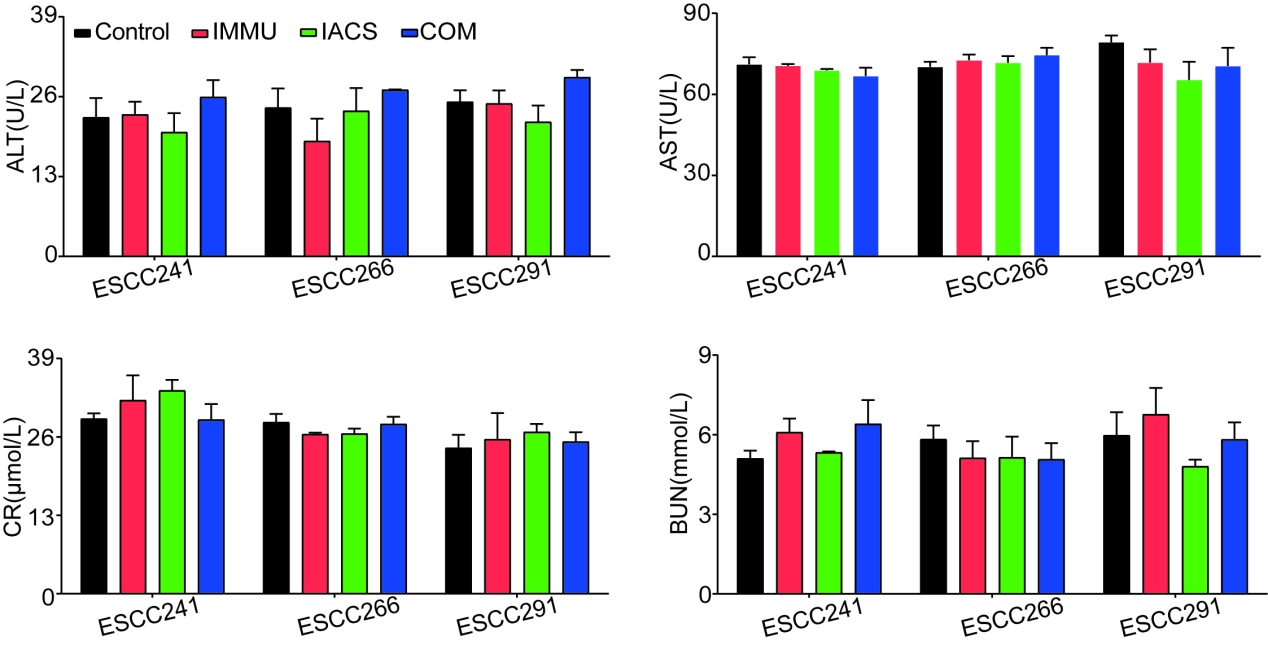


**Supplementary Figure 9. The effects of combination therapy of IMMU and IACS on liver and renal function in ESCC PDX models.** The serum levels of liver and renal function index enzymes ALT, AST, Crea, and BUN in mice were detected after combination treatment. More 4 replicate samples were averaged, and the error bars represent the SEM.
