# Supplementary material for: A TROP2-targeting ADC synergizes with oxidative phosphorylation inhibitor to enhance apoptosis in ESCC by suppressing the PI3K-AKT-mTOR signaling pathway
Source: Cell Death Dis. 2025 Dec 1;17(1):67. doi: 10.1038/s41419-025-08278-5 (PMC12827259; doi:10.1038/s41419-025-08278-5)

Full uncropped Gels and Blots images

Full unedited gel for Figure _1C

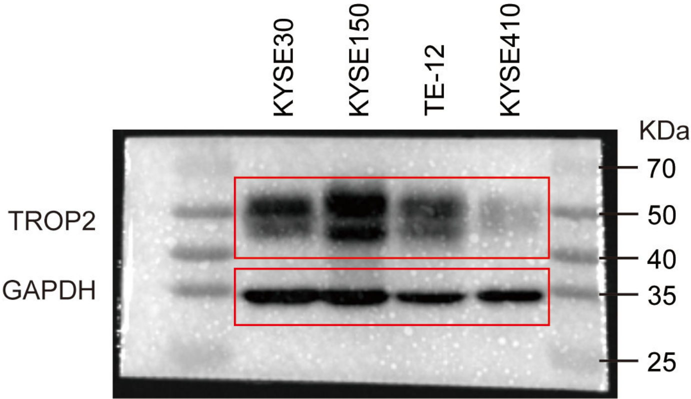

Full unedited gel for Figure _5E

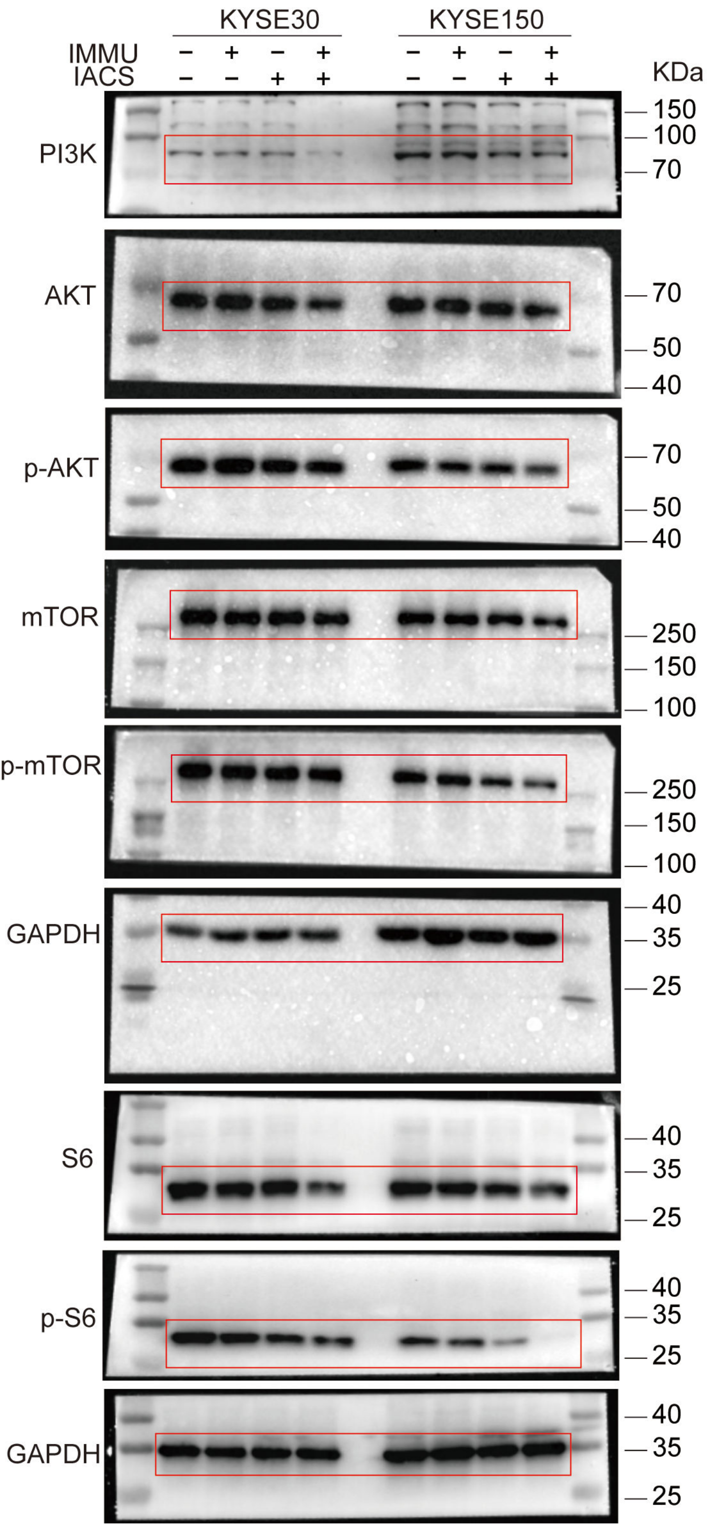

Full unedited gel for Figure _5G

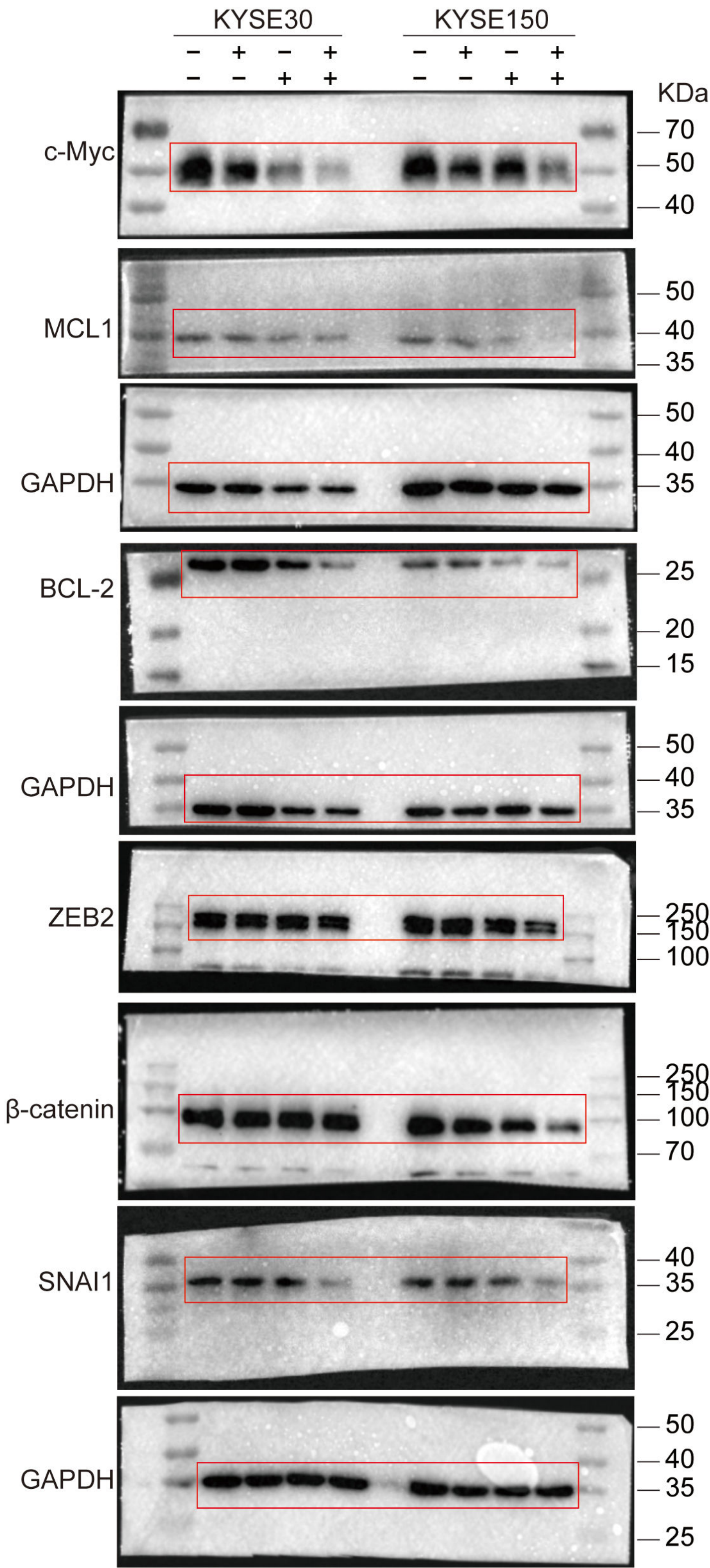

Full unedited gel for Figure _6A

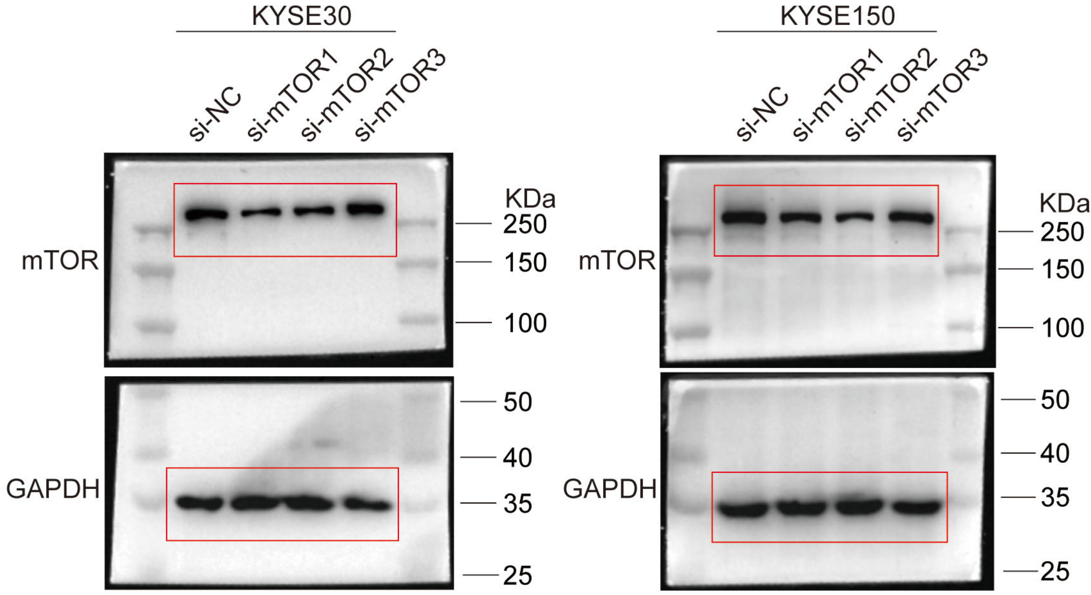

Full unedited gel for Figure _6E

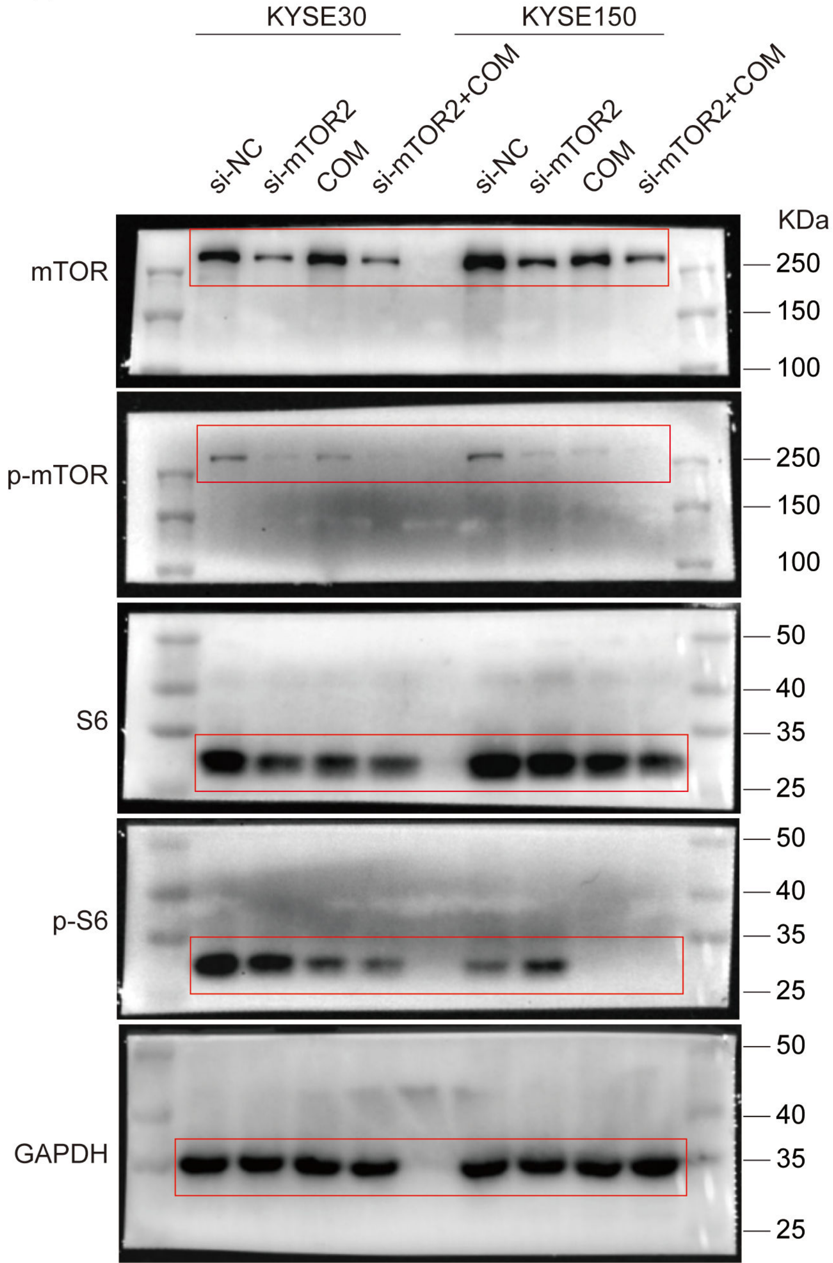

Supplement: Supplementary file 2 — SUPPLEMENTAL MATERIAL [file 41419_2025_8278_MOESM2_ESM.pdf]
